# Evolution characteristics and policy implications of new urbanization in provincial capital cities in Western China

**DOI:** 10.1371/journal.pone.0233555

**Published:** 2020-05-26

**Authors:** Zou Ya-Feng, Deng Min, Li Ya-Jing, Rong Yao

**Affiliations:** 1 School of Public Administration, Inner Mongolia University, Hohhot, China; 2 Institute of Geographic Sciences and Natural Resources Research, CAS, Beijing, China; 3 School of Resource and Environment Science, Wuhan University, Wuhan, China; Shandong University of Science and Technology, CHINA

## Abstract

New urbanization is the fundamental approach to achieve the healthy, stable, and sustainable development of the Chinese economic society. It is also the basic outlet to eliminate the “dual economic structure” in urban and rural areas. Based on the connotation of new urbanization, we constructed an evaluation system using population development, economic development, quality of life, infrastructure, resources and environment, and urban and rural harmonious development. The entropy and weighted summation methods were used to measure the level of new urbanization for 11 provincial capital cities from 2005 to 2018, and policy implications were analyzed correspondingly. The results show that there are significant differences in the development levels of new urbanization in these cities, with infrastructure construction being the primary driver. These developments have placed the economy and environment under great pressure. The quality of urban life and the level of infrastructure construction need to be improved because of the expanding economic gap between urban and rural areas. These cities with poor internal coordination also have apparent differences amongst individual factors. Overall, the policies on these factors play a positive role in the process of new urbanization. In the future, provincial capital cities need to consider the weak links and provide more focus on employment and education.

## 1. Introduction

Urbanization is a significant driving force for human progress and economic development and is an important symbol to measure the social and economic development of a country or region [1–3]. Since China’s reform and the opening of its economy, its urbanization rate has increased from 17.92% in 1978 to 57.35% in 2016 [4, 5]. With rapid urbanization, the national economy has developed effectively, and the living standards of urban and rural residents have been drastically improved [6, 7]. However, substantial and severe problems, such as urban-rural dual economic structures, unreasonable layout of urban spaces, and extensive urban development are severe [8, 9]. Numerous studies have noted that urbanization based on the “city-biased policy” in China is of low quality as it leads to resource waste and a larger developmental gap between urban and rural areas [10–15]. Recently, the phenomenon of “ghost cities” across China suggests there are wasted urban resources on an entirely new scale [16, 17]. However, the path of urbanization in China has been distinctive from other countries, which means China cannot directly implement their models for urbanization [7, 12].

To address the challenges above, it is inevitable for China to pursue a new type of urbanization that places more emphasis on internal quality improvements and coordinated developments of urban and rural areas [7, 18, 19]. The Chinese government formally put forward the idea of “taking the road of urbanization with Chinese characteristics” in 2002, and explicitly used the term “new urbanization” at the 18th National Congress of the Communist Party of China (CPC) in 2012. In March 2014, China’s National Development and Reform Commission proposed the *National New Urbanization Plan (2014–2020)* (NNUP), which establishes a blueprint for China’s future urbanization and economic development [20]. The plan stresses people-oriented urbanization and focuses on the key strategic aims to comprehensively improve the urbanization quality, optimize the layout and form of urbanization, enhance a cities’ livability, promote the orderly citizenization of rural migrant populations and urban-rural integration [21, 22]. And in the same year, the pilot work began. By 2016, a total of two provinces (Anhui and Jiangsu) and 246 cities (towns) in China were listed as pilot projects in three batches. Then, *the Key Tasks of the New Urbanization Construction in 2019* were released, with emphasis on speeding up the citizenization of the agricultural transfer population, improving the quality of urban development and accelerating urban-rural integration.

As a new concept proposed by the Chinese government, new urbanization has not been clearly defined. In 2012, the report of the 18th CPC National Congress pointed out that the “new” part of new urbanization is urban-rural coordination, integration, and interaction. It entails intensive economic, ecological viability, and harmonious growth, and also represents the coordinated development of large, medium, and small cities, small towns, and new rural communities. Immediately, scholars expounded the connotation of new urbanization from the following aspects: the relationship between urban and rural areas, the relationship among cities of different scales, ecological construction and sustainable development [23–25]. However, they did not pay enough attention to the role of people in the new urbanization until Premier Li stressed “carrying out a new people-oriented urbanization” as a key task for 2014 in the 12th National People's Congress [6]. Since then, based on the people-oriented idea, the academic circles further refined new urbanization as people-oriented urbanization [26, 27], equalization of public service [2, 22], urban livability [28, 29], urban-rural integration [30, 31], ecological protection [32, 33], resource-saving [34, 35], and sustainable development [25]. Overall, traditional urbanization in China is characterized by high resource consumption, high carbon output, high pollution, low efficiency, and urban-rural imbalance. In contrast, the new urbanization is the process that emphasizes high efficiency and low carbon output of economy, ecologic and environmental protection, urban-rural integration, infrastructure and quality of life improvement. Its core is to be “people-oriented,” and its essence is to constantly improve the quality and connotation of urbanization construction [36, 37].

The exploration and practice of a characteristic town (*tesexiaozhen*) from Zhejiang provide a vital way for China to approach new urbanization [38, 39]. A low carbon eco-city and urban agglomerations have also begun to be widely described [40–42]. China’s new urbanization has achieved some achievements regarding the eco-construction and reform of household registration systems (*hukou*); yet, there are still many challenges, especially in the less-developed economic regions, western China for example [43]. The division of China into east, central and western regions, began in 1986 with the Seventh Five-Year Plan for National Economic and Social Development of the People's Republic of China. The division was based on the function of policy, not administration or geography. As the eastern region benefited the earliest from the opening-up policy, its level of economic activity is relatively high. The central region is less developed, and the western area is least. Two pilot provinces of new urbanization, Jiangsu and Anhui, are located in eastern and central China, respectively. Besides, the idea of the characteristic town (*tesexiaozhen*) originated from the eastern region, too. However, the western region has provided energy and resource services for the rest regions in China for a long time [44]. Until the proposal of the Western Development Strategy in 2000, a strategic task of the Chinese government to speed up the development of the western region, the development of the western region was put in an important position. Compared with the central and eastern regions, the western region lies inland with the sparsely populated and fragile ecological environment, while it has the advantages of resource and is also a gathering area of ethnic minorities in China. They lead to the different industrial structures and lifestyles, as well as different rates, character, sustainable development problems and challenges of new urbanization in western China [22]. This means that the pilot experience of new urbanization in the central and eastern regions cannot be fully applied to the western regions. During this critical period, strengthening comprehensive research on new urbanization levels is of great significance for the development of healthy urbanization in China.

In recent years, the new urbanization in China has attracted widespread international attention. Of the hundreds of articles covering this theme, the research contents have mainly focused on the essential connotation and development characteristics of new urbanization [2, 26, 45], the establishment of index systems and quality evaluations of new urbanization [5, 20, 46–49], the relationships between new urbanization and new industrialization [41, 50], and intensive land-use [51, 52]. Generally, there has been little research on the comprehensive measures of new urbanization and the driving factors, especially the policy implications, for differences in urbanization development. In addition, the research scope has mainly been concentrated at the national or provincial level [22, 25] and developed cities or regions [47, 53], which are located in the central and eastern parts of China, while less focus has been on western provincial capitals. There have been more static studies on the spatial dimensions [47, 54], but dynamic changes with time have been relatively scarce. Throughout the history of China’s urbanization, achievements and challenges have been closely related to national policies; this is also the main reason why China’s urbanization differs from other countries and presents distinct stages. Based on its special development conditions and important strategic position, the western region's local policies also play a significant role in the process of urbanization. Therefore, to objectively and comprehensively evaluate the level of new urbanization, this study constructed a more comprehensive index system based on the connotation of new urbanization. The entropy and weighted summation methods were used to calculate the index weight and total score to analyze the driving factors in 11 provincial capital cities from 2005 to 2018. Meanwhile, we analyzed the policy implications of each period correspondingly. This study was performed with the aim of providing theoretical support and guidance to promote new urbanization construction and urban-rural integration developments in western China.

## 2. Materials

### 2.1. Study area

**Fig 1:**
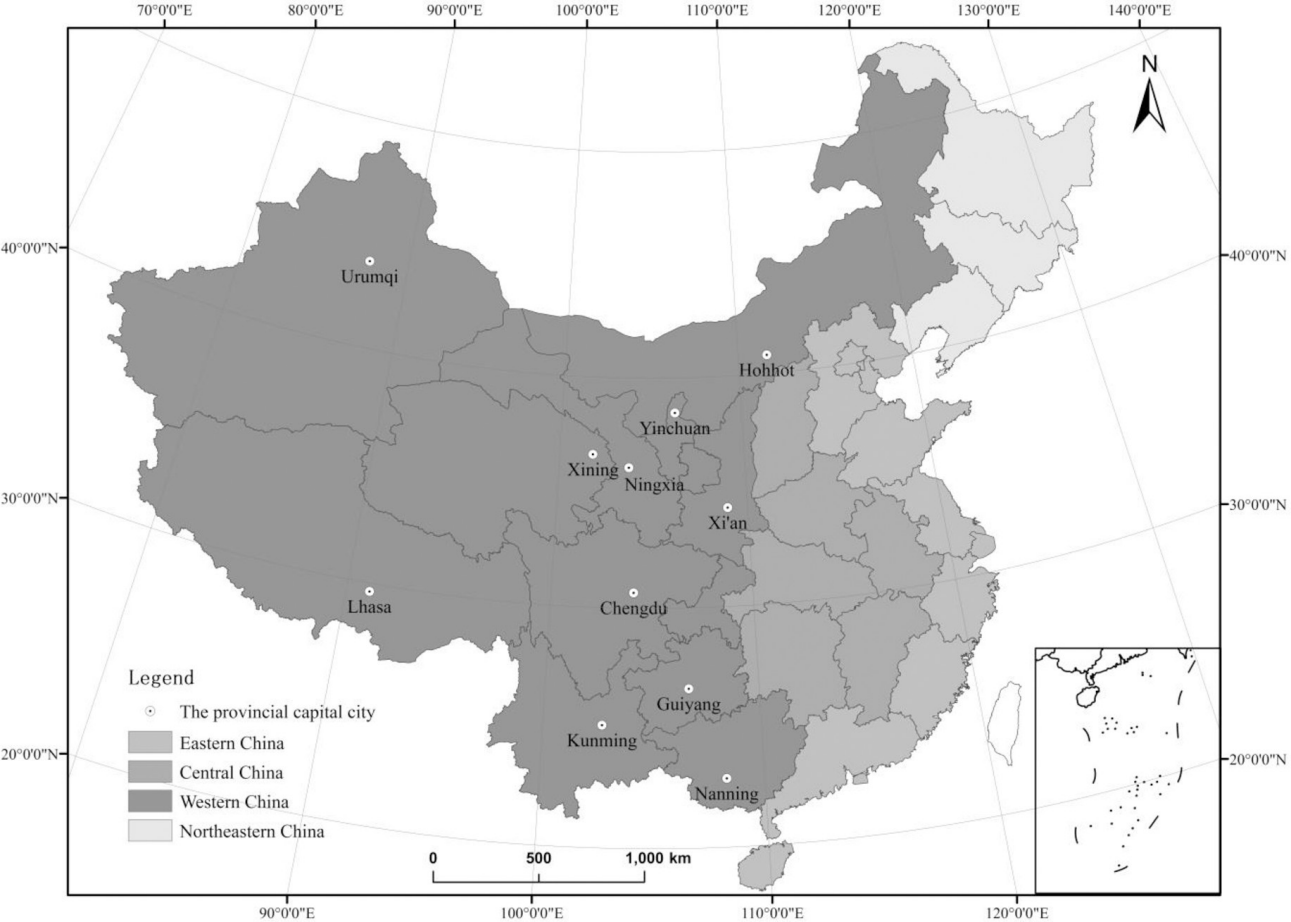
Study area of Western China.

Western China (Fig 1) includes one municipality (Chongqing), five autonomous regions (Xinjiang, Tibet, Ningxia, Inner Mongolia, and Guangxi), and six provinces (Qinghai, Gansu, Shaanxi, Sichuan, Yunnan, and Guizhou). Due to the specificity of the administration, the level of economic development, and its size, population, and impact, Chongqing is not included in this study. The 11 provincial capital cities are Chengdu (the capital of Sichuan), Kunming (Yunnan), Guiyang (Guizhou), Xi’an (Shaanxi), Lanzhou (Gansu), Xining (Qinghai), Lhasa (Tibet), Urumqi (Xinjiang), Yinchuan (Ningxia), Hohhot (Inner Mongolia), and Nanning (Guangxi). As one of the aridest areas in the mid-latitudes, Western China features a complex terrain, a vulnerable ecological environment and is landlocked. The area of the western region accounts for 71% of the country, while the population is only 27% of the country (2018). In addition, its economic performance lags behind the rest of China [55, 56]. China's regional economic development is uneven, not only among the east, central and western regions, but also within each province [57, 58]. Due to the obvious siphon effect of western capital cities, their development level is far ahead of other cities in the corresponding province. Since 2014, most western provincial capitals (districts) had been gradually listed as new urbanization pilots in three batches, making them the pioneers in implementing the new urbanization strategy. These cities are the impetus of regional development, which is also the reason why we choose them as study areas.

### 2.2. Data sources

This research selected 11 provincial capitals in the western region of China as the basic evaluation unit, taking 2005, 2010, 2015 (three closing years of the 10^th^, 11^th^, and 12^th^ Five-Year Plans of China, respectively), and 2018 (the latest time for data available) as the evaluation time nodes. These years can better reflect the construction results during each Five-Year Plan period. The 10^th^ Five-Year Plan is the first Five-Year Plan after the Western Development Strategy (2000) was put forward. Then the *11*^*th*^ *Five-Year Plan for the development of the western region* was adopted. During the 12^th^ Five-Year Plan period, the term “new urbanization” was formally proposed (2012), NNUP was issued (2014), and the first pilot list was released (2014). For western China, these three Five-Year Plans are the period for major significant development opportunities. And the year of 2018 was chosen to reflect the phased achievements of new urbanization in the western region during the 13^th^ Five-Year Plan period (2016–2020) to a certain extent.

To ensure accuracy and comparability, the data were all derived from the statistical yearbook and statistical bulletin of the corresponding years from the national and provincial statistics bureaus, mainly including the China Statistical Yearbook, China Urban Statistical Yearbook, China’s environmental situation bulletin and statistical yearbooks of various cities. Due to the lack of some data in Lhasa, the data were interpolated after processing those for adjacent years.

## 3. Methodology

**Fig 2:**
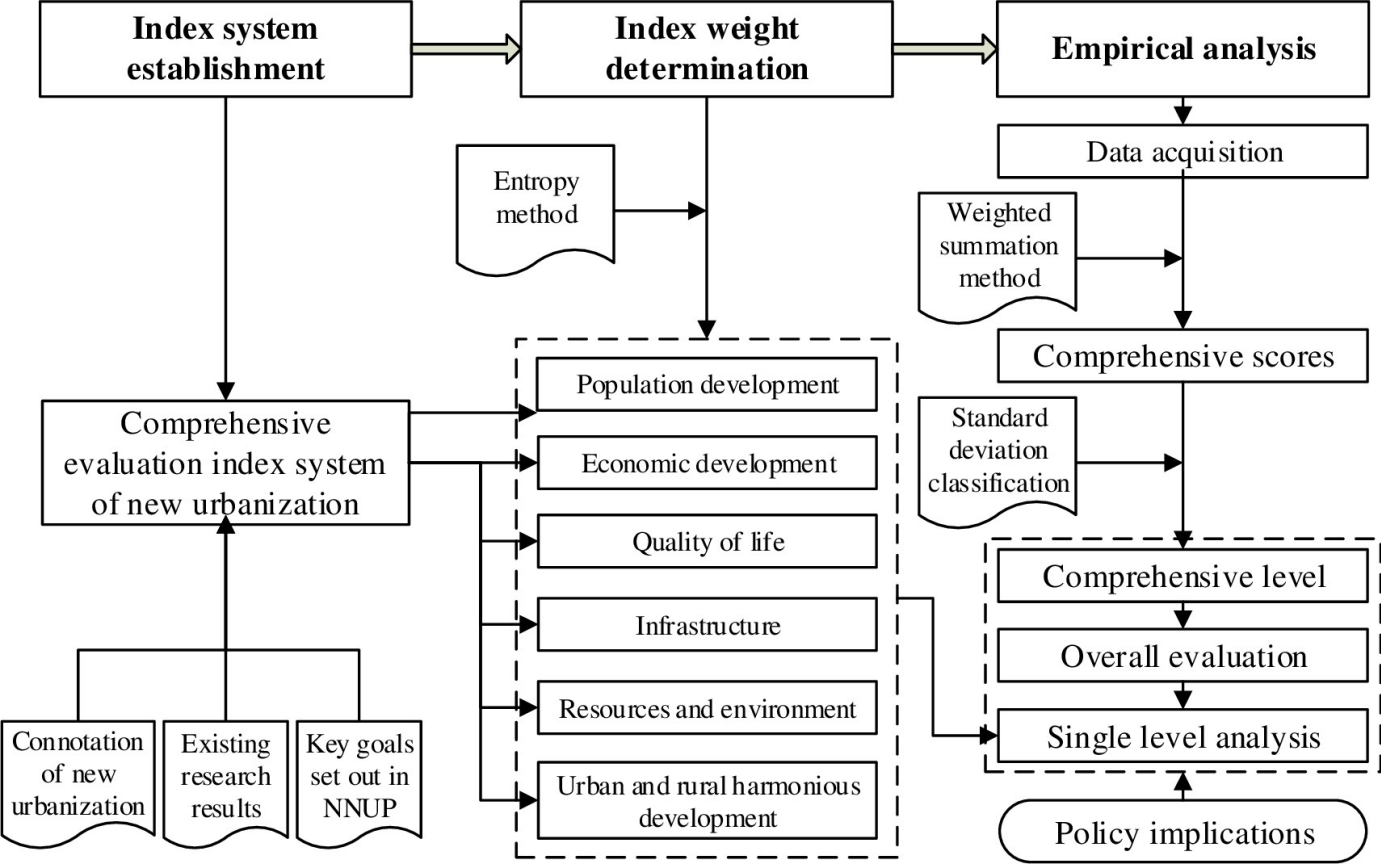
Research flowchart.

Inspired by recent research on new urbanization and key goals established in the NNUP, this study established a multi-system and multi-level comprehensive evaluation index for new urbanization. The index weight was determined using the entropy method, and the new urbanization comprehensive scores of the western provincial capitals were calculated using the comprehensive index method. The standard deviation classification was chosen to divide these cities into three levels (high, medium, and low), and the spatial differences of the urbanization development were analyzed with the ArcGIS software. Geared towards differences at the monomial level of new urbanization for western provincial capitals, this research dynamically compared six perspectives: population development, economic development, residents’ quality of life, infrastructure, resources and environment, and urban and rural harmonious development. The main drivers of these differences, especially the influence of policies, were then analyzed in detail (Fig 2).

### 3.1. Established index system

In order to evaluate the results of implementation of the new urbanization, the existing researches have carried out qualitative evaluation from the aspects of the problems [2], the route to realization [6, 27], the potential impacts [59], or constructed some index systems to carry out quantitative measurement [20, 22, 60]. Different index systems are different understandings of the new urbanization concept in origin. The existing research on the definition of the concept mainly considers three aspects: urban development with humans as the core, urban-rural integration, and environmental protection [2, 20, 22, 60]. With the improvement of the connotation and quality of new urbanization construction, the index system should be more comprehensive.

This study draws lessons from existing research results [5, 45–49] and the key goals set out in the NNUP, especially the concept of “people-oriented” urbanization. The study also took full account of the actual development in the western region. This research selected six criterion layers and 24 indicators to measure the comprehensive level of new urbanization based on the concept and characteristics of new urbanization and according to the principles that the index system should be representative, comprehensive, scientific, systematic, and operable (Table 1).

**Table 1:** Comprehensive evaluation index system for new urbanization.

Target layer	Criterion layer	Index layer	Effect direction	Unit
Comprehensive development level of new urbanization in provincial capital cities in Western China (X)	Population development (X_1_)	Permanent resident urbanization rate (X_11_)	positive	%
		Proportion of non-agricultural employees (X_12_)	positive	%
		Number of college students per 10,000 people (X_13_)	positive	people
	Economic development (X_2_)	Per capita GDP (X_21_)	positive	CNY
		Proportion of output value of tertiary industries (X_22_)	positive	%
		GDP growth rate (X_23_)	positive	%
		Total imports and exports per capita (X_24_)	positive	Dollar
	Quality of life (X_3_)	Per capita disposable income of urban residents (X_31_)	positive	CNY
		Per capita consumption level of urban residents (X_32_)	positive	CNY
		Registered unemployment rate of urban residents (X_33_)	reverse	%
		Number of urban workers participating in endowment insurance (X_34_)	positive	ten thousand people
		Number of urban workers participating in medical insurance (X_35_)	positive	ten thousand people
		Broadband internet access subscribers (X_36_)	positive	ten thousand households
	Infrastructure (X_4_)	Owned bus vehicles per 10,000 people (X_41_)	positive	vehicles
		Owned medical beds per 10,000 people (X_42_)	positive	beds
		Owned public library collection per 100 people (X_43_)	positive	books
		Urban road area per capita (X_44_)	positive	m^2^
	Resources and environment (X_5_)	Urban construction land per capita (X_51_)	reverse	m^2^
		Green coverage rate in built-up area (X_52_)	positive	%
		Proportion of harmless treated garbage (X_53_)	positive	%
		Wastewater treatment rate (X_54_)	positive	%
		Proportion of good air quality days in cities (X_55_)	positive	%
	Urban and rural harmonious development (X_6_)	Ratio of per capita disposable income between rural and urban residents (X_61_)	positive	
		Ratio of per capita consumption between rural and urban residents (X_62_)	positive	

The first criterion layer is population development, which is reflected as the population shifting from rural areas to cities, agricultural population transformations to non-agricultural, and the continuous improvements in population quality. The second is economic development as embodied in economic agglomeration and the efficient development, optimization, and upgrades for industrial structures. The third is quality of life. The new urbanization adheres to the “people-oriented” urbanization, which means it ensures and improves people’s livelihoods and gives top priority to improving people’s quality of life. This can be accomplished by increasing the disposable income of urban residents and by expanding the coverage of endowments and medical insurance. The fourth is infrastructure, as urban development requires infrastructure support. Strengthening the infrastructure construction and improving urban functions are the objective requirements of new urbanization. The Fifth is the rigid constraints of resources and environment for urban development. New urbanization advocates “resource-savings” and “environment-friendly,” to promote sustainable urbanization development. The sixth is urban and rural harmonious development, as reflected in the narrowing wealth gap between urban and rural residents. This is focused on raising farmers’ incomes and promoting their ability to consume.

### 3.2. Comprehensive evaluation model: Entropy method

The entropy method is an objective weighting approach that determines the index weights according to the information provided from the observed value of each index. A smaller index entropy value suggests its degree of variation is greater and provides more information. Correspondingly, in the comprehensive evaluation, a larger weight indicates it has a greater role and vice versa [47, 61, 62]. The entropy value provides the degree of variation for each index value, and the weights of the corresponding years can be calculated. The main steps are as follows:

To eliminate the interference of dimensions, order of magnitude differences, and the influence of the positive or negative orientation of the indicator on the results, the initial data should be standardized.

First, the initial data are standardized by:

$$Positive\;index:\;X_{ij}^{'}=\frac{X_{ij}-\min\{X_{j}\}}{\max\{X_{j}\}-\min\{X_{j}\}}.$$ (1)

$$Reverse\;index:\;X_{ij}^{'}=\frac{\max\{X_{j}\}-X_{ij}}{\max\{X_{j}\}-\min\{X_{j}\}}.$$ (2)

where *X*_*ij*_ is the original value of the *j*th index in the *i*th city, and $X_{ij}^{'}$ is the standardized value (*i* = 1, 2,…, m; *j* = 1, 2,…, n). Second, the index information entropy is calculated by:

$$e_{j}=-k\sum_{i=1}^{m}\left(Y_{ij}\times \ln Y_{ij}\right),$$ (3)

with

$$k=\frac{1}{\ln m},$$ (4)

$$Y_{ij}=\frac{X_{ij}^{'}}{\sum_{i=1}^{m}X_{ij}^{'}}.$$ (5)

where *Y*_*ij*_ is the proportion of city *i* in indicator *j* and *k* is a constant.

Third, the indexes are weighted:

$$w_{i}=\frac{1-e_{j}}{\sum_{j=1}^{n}(1-e_{j})}.$$ (6)

where 1−*e*_*j*_ is the redundancy of the information entropy.

Finally, the weighted summation method is used to calculate the total score of each city as:

$$S_{i}=\sum_{j=1}^{n}\left(w_{i}\times X_{ij}^{'}\right).$$ (7)

where *S*_*i*_ is the comprehensive level score of new urbanization in the city *i*, *w*_*i*_ is the weight of the *i*th index, and $X_{ij}^{'}$ is the standardized value of the *j*th index in the *i*th city.

### 3.3. Comprehensive level classification - standard deviation classification

Standard deviation classification is an unequal classification method. The classification number is determined from the degree of the data dispersion and the multiple of the adopted standard deviation [63]. It is commonly used to represent differences between values and averages. This study divided the new urbanization levels of the western provincial capitals into three levels for 2005, 2010, 2015, and 2018 using the standard deviation classification. The formula is as follows:

$$L(S_{i})=\left\{ \begin{array}{l} H,\;(\bar{S}+0.5\delta ,1]\\ M,\;[\bar{S}-0.5\delta ,\bar{S}+0.5\delta ]\\ L,\;[0,\bar{S}-0.5\delta ) \end{array}\right.,$$ (8)

with

$$\bar{S}=\frac{1}{m}\sum_{i=1}^{m}S_{i},$$ (9)

$$\delta =\sqrt{\frac{1}{m}\sum_{i=1}^{m}{\left(S_{i}‑\bar{S}\right)}^{2}}$$ (10)

where *L*(*S*_*i*_) is the new urbanization level of the *i*th city, *H* is the high level, *M* is the medium level, *L* is the low level, $\bar{S}$ is the average composite score of the new urbanization level for the 11 cities, and *δ* is the standard deviation.

## 4. Results

### 4.1. Weight determination

As the dimensions and orders of the magnitudes for each index are inconsistent, the min-max normalization method (Formulas (1) and (2)) was used to standardize the index values, and the entropy method (Formula (4)) was used to determine the weights of each index in the corresponding year. The weights were then used to calculate the criterion factor layer weights by adding the index layer (Table 2).

**Table 2:** The weights for the comprehensive evaluation index system on new urbanization.

Target layer	Weight	Criterion layer	Weight				Index layer	Weight			
			2005	2010	2015	2018		2005	2010	2015	2018
Comprehensive development level of new urbanization in provincial capital cities in Western China (X)	1	Population development (X_1_)	0.113	0.115	0.114	0.117	X_11_	0.038	0.039	0.038	0.039
							X_12_	0.034	0.034	0.034	0.035
							X_13_	0.041	0.043	0.042	0.043
		Economic development (X_2_)	0.182	0.179	0.177	0.161	X_21_	0.042	0.046	0.044	0.038
							X_22_	0.044	0.042	0.038	0.038
							X_23_	0.048	0.043	0.050	0.037
							X_24_	0.048	0.049	0.045	0.048
		Quality of life (X_3_)	0.265	0.260	0.260	0.280	X_31_	0.043	0.046	0.041	0.042
							X_32_	0.040	0.039	0.035	0.038
							X_33_	0.048	0.044	0.037	0.053
							X_34_	0.045	0.044	0.047	0.054
							X_35_	0.043	0.045	0.047	0.049
							X_36_	0.047	0.042	0.053	0.044
		Infrastructure (X_4_)	0.175	0.180	0.184	0.162	X_41_	0.048	0.045	0.042	0.038
							X_42_	0.041	0.052	0.042	0.035
							X_43_	0.041	0.038	0.042	0.041
							X_44_	0.044	0.045	0.058	0.048
		Resources and environment (X_5_)	0.190	0.187	0.180	0.191	X_51_	0.034	0.035	0.035	0.037
							X_52_	0.043	0.037	0.036	0.037
							X_53_	0.036	0.039	0.034	0.040
							X_54_	0.039	0.042	0.037	0.038
							X_55_	0.038	0.035	0.038	0.039
		Urban and rural harmonious development (X_6_)	0.075	0.078	0.085	0.090	X_61_	0.037	0.041	0.043	0.051
							X_62_	0.038	0.037	0.042	0.039

From the overall data size of the criterion layer, the weights for the quality of life in the four time nodes are the highest. In contrast, the weights of the urban and rural harmonious development are the lowest. From the trends in the sizes of the four time nodes, as the economic development, the weights for the quality of life, and the resources and environment decrease, the weights for the infrastructure and the urban and rural harmonious development increase. Only the weights for the population development remain the same over the periods analyzed. This shows that as the influence of the previous indicators declines, the influence of the latter three categories is constantly increasing or remains unchanged.

## 4.2. Overall evaluation

**Fig 3:**
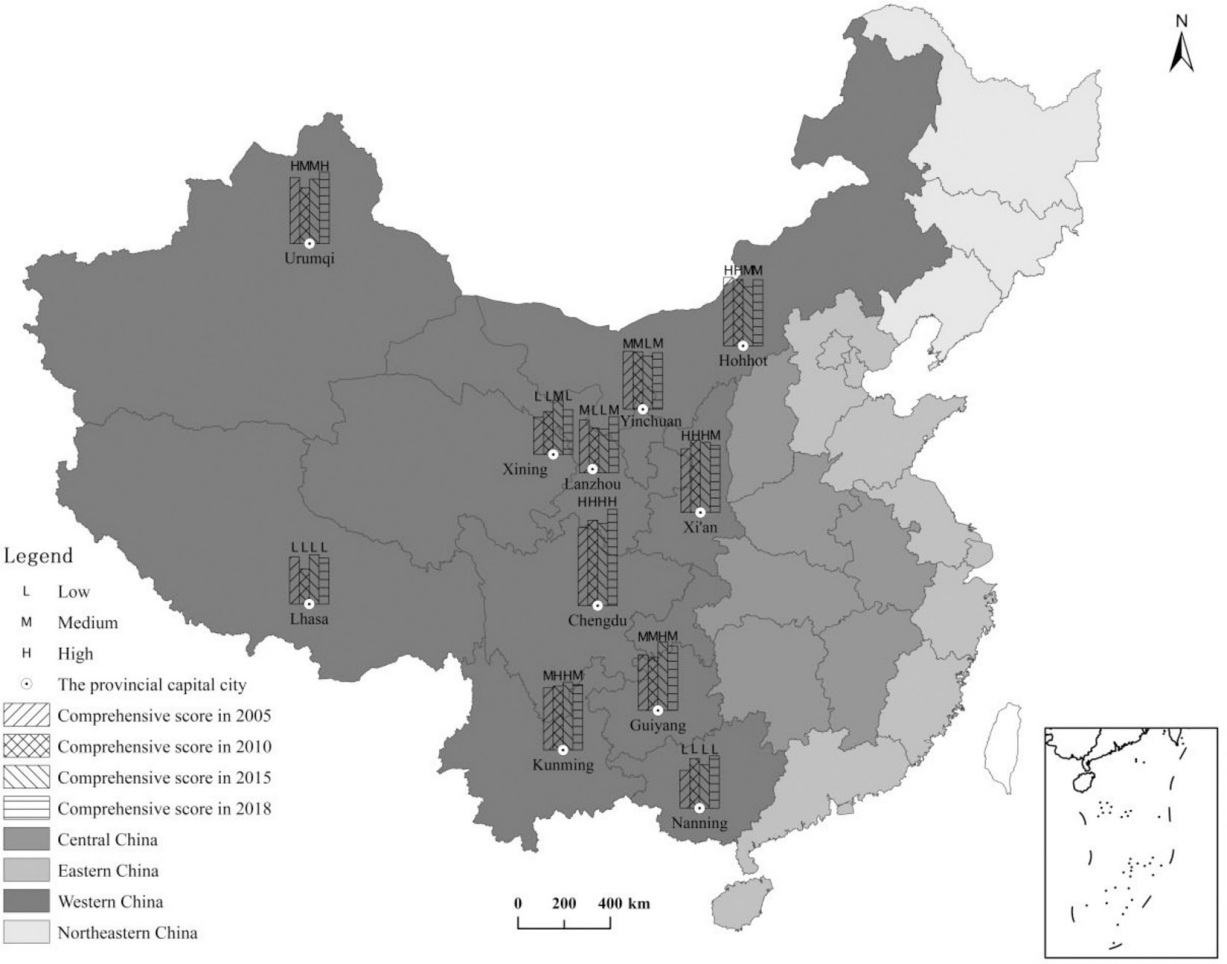
Spatial distribution of the new urbanization comprehensive level in provincial capital cities of Western China.

The urbanization comprehensive level calculation of Formula (7) was adopted to calculate the comprehensive scores for the urbanization development level of the 11 provincial capital cities in Western China from 2005, 2010, 2015, and 2018. The new urbanization levels of each city were then divided into three levels using the standard difference method. The spatial distribution map for the development levels was plotted in ArcGIS (Fig 3), and the final results are as follows:

1. Distinctively uneven developments exist in different cities. Overall, the development levels of new urbanization in the cities for the different periods were quite different. From 2005 to 2018, Chengdu, Kunming, and Xi’an were classified with a high level, with Chengdu scoring the highest (0.595, 0.651, 0.625, and 0.734, respectively), which was quite different from the other cities. This is because Chengdu is the scientific, commercial, and financial center of Southwest China and an important high-tech industrial base of the country. Due to its special location of low latitude and high altitude, Kunming is an important tourist and commercial city in China. It has abundant resources, a good living environment, and great development capabilities. In Xi’an, the level of new urbanization has been steadily increasing. As a famous historical and cultural city and an important base of scientific research, education, and industry, Xi’an has a sound economic foundation. With an expanded openness to the outside world, its comprehensive strength has been continuously enhanced. However, Lanzhou, Xining, Nanning, and Lhasa have developed at low levels during the four time nodes. Limited by its location and resource constraints, Lanzhou’s industrial structure is relatively backward, and its economic development lacks momentum. Restricted by its industrial structure, the economy of Xining also lags and the income levels of the urban and rural residents are lower. Nanning lacks policy and financial support, and its transportation and other infrastructure is inadequate. Its level of industrial modernization is low, and the motive force for urban development is insufficient. Lhasa is located in the southwestern frontier of China. The special history and poor natural resources have led to its weak economic base, sparse population, poor industrial structure, and low level of education. Due to the influence of the geographic and humanistic factors, the level of new urbanization in Tibet is low and its progress is slow, so it has always been in a relatively backward state. The scores for the other cities vary in different degrees, which further reflect the significant regional differences in the new urbanization status of western provincial capitals due to different locations, resource conditions, and economic bases.
2. The urbanization quality has been continuously improved. According to the mean weight of the factors, the quality of life accounted for the highest weight (0.266), followed by resources and environment (0.187) from 2005 to 2018. This indicates that the quality of life for residents is the primary driving force of new urbanization processes in the western provincial capitals over the past 14 years of the research period. This also reflects that “people-oriented” urbanization is the core of new urbanization activities, which emphasizes the overall improvements to the internal quality over the traditional development model. This pushes the urbanization construction developments towards a greener direction while saving resources and protecting the environment.
3. Urban development has lacked internal coordination. From the comprehensive scores of the same period, the levels of new urbanization in different cities had different distributions. For example, compared with other provincial capitals in 2005, Lhasa scored the highest in infrastructure (X_4_, 0.135) and lowest in population development (X_1_, 0.007) and urban and rural harmonious development (X_6_, 0.001). In 2010, Urumqi had the best level of urban and rural harmonious development and infrastructure conditions (X_6_, 0.078 and X_4_, 0.098, respectively), but was worst in resources and environmental conditions (X_5_, 0.054). In 2015, Lanzhou scored the highest in population development (X_1_, 0.114), but ranked last in infrastructure and resources and environment (X_4_, 0.021 and X_5_, 0.061, respectively). In 2018, Lanzhou still has the highest score of population development (X_1_, 0.115), while has the lowest score of resource environment and urban-rural harmonious development (X_5_, 0.080 and X_6_, 0.009, respectively). Thus, it can be seen that the internal developments of these cities are uncoordinated.

### 4.3. Comparative analysis for the single level of new urbanization in capital cities of Western China

#### 4.3.1. Population development

Except for the significant increase at Lanzhou and the decrease at Xi’an from 2005 to 2018 and the increase at Kunming between 2010 and 2018, the overall level of population development in the other cities did not change markedly during the study period. In 2005, Xi’an scored the highest (0.112), followed by Lanzhou (0.099) and Guiyang (0.103). In 2010, the top three were Lanzhou (0.108), Xi’an (0.107), and Hohhot (0.089). In 2015, Lanzhou and Xi’an continued to rank first and second (0.114 and 0.094, respectively), while Guiyang ranked fourth in 2010 (0.086), and returned to third place in 2015 (0.089). In 2018, Lanzhou, Guiyang and Xi'an are still in the top three (0.115, 0.094, and 0.087, respectively). Lhasa scored the lowest over these three years.

Lanzhou has maintained a high level of population development throughout the study period. This is because the proportion of people with rural household registrations was large in the permanent urban population of Lanzhou, and most of these individuals moved into cities to work and settle. In addition, the boundary between the urban and suburban regions has become increasingly blurred with the high-intensity urban renewal and urban construction in Lanzhou in recent years; thus, the statistics of the urban population are not very accurate. However, due to the impact of the fractional line and enrollment policy, the number of college students per 10,000 people in Lanzhou was significantly higher than the other cities. However, Xi’an does not have such an advantage. In addition, the economy of Xi’an has been backward, and its competitiveness is not as good as that for the central and eastern cities. Therefore, although a large number of talents have been trained there, most of them have outflowed to other regions.

Kunming reformed the household registration system beginning in 2010. The city comprehensively applied the unified household registration system between the urban and rural areas and the process of “*Nongzhuangcheng*” (rural population transfer to urban residents). While promoting the rapid development of the social economy, Kunming has absorbed a large number of people to move in, which greatly improved the level of urbanization.

Most noteworthy, Lhasa is located in the southwest frontier of China. Its special historical and natural geographical environment has led to a weak economic foundation, unreasonable industrial structure, and low level of education. This is also one of the main reasons for the slow development of urbanization over the entire Tibet region.

**Fig 4:**
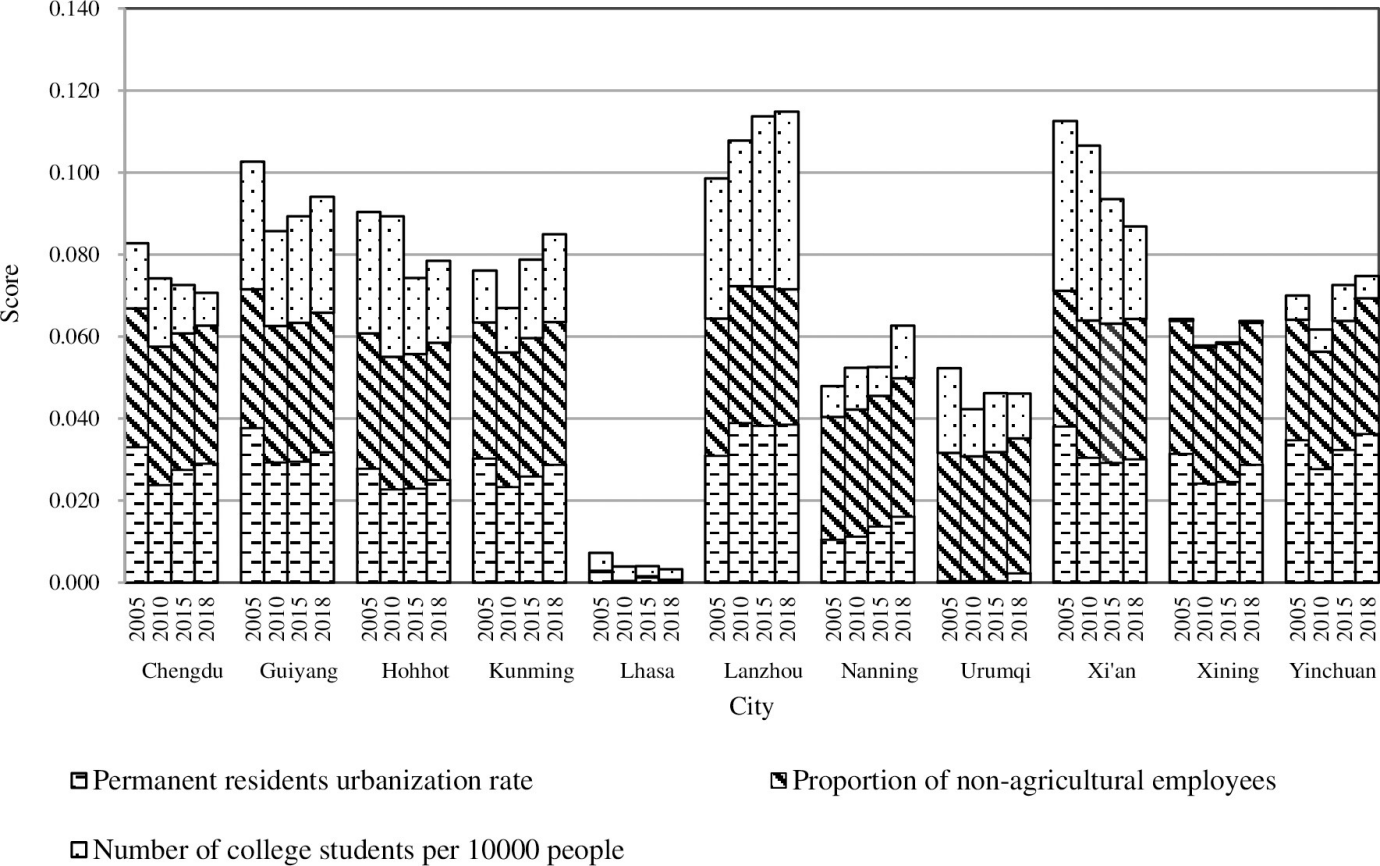
The “population” of new urbanization in the provincial capital cities of Western China.

In the specific indicators shown in Fig 4, the urbanization rate for permanent residents was relatively uniform among these cities from 2005 to 2018, and the proportion of non-agricultural employees grew slowly. However, there was a large gap in the number of college students per 10,000 people, which became the most important influencing factor in the development of the urban population. Overall, the above reflects that the employment situation for the secondary and tertiary industries in cities was very poor due to the limited number of jobs and the unbalanced allocation of educational resources among the cities. To promote future urbanization, more attention should be given to employment and education.

#### 4.3.2. Economic development

The state of economic development for most of these cities is not stable. With the exception of Urumqi, which has been in the top three from 2005 to 2018, the other cities have experienced varying degrees of ups and downs. The cities with the highest scores in 2005 and 2015 were Hohhot (0.120) and Urumqi (0.098), and Chengdu scored the highest in 2010 and 2018 (0.101 and 0.127, respectively), while Nanning has always been ranked lower, which was closely related to the economic development structure and pattern of each city over the different years.

Urumqi ranked second in 2005, first in 2010 and 2015, and third in 2018 regarding economic development, indicating the city’s economy is relatively good. In 2005, Urumqi seized the opportunity of the fiftieth anniversary of the establishment of the autonomous region to conduct various forms of tourism promotional activities. The development of tourism has effectively led to the rapid development of related industries. During the 11th Five-Year Plan period, Urumqi vigorously promoted the modern industries with these characteristics, which maintained sustained economic growth. Subsequently, with the development of the new Western Development and the Belt and Road, Urumqi achieved a significant economic development due to its unique location and the leading role of the core area of the Silk Road Economic Belt.

Chengdu’s economy is the most volatile, ranking fourth in 2005, first in 2010, and seventh in 2015, while in 2018, return to first place. Chengdu has a large labor force, relatively concentrated resources, and an excellent economic base. Since 2010, the government of Chengdu has vigorously supported the development of private economies and emerging industries through policies, which has absorbed a large amount of the labor market and stimulated a considerable increase in the economy. Subsequently, Chengdu’s economy has continued to maintain a stable growth, but the momentum has been slightly inadequate. Although the economic score fell to seventh place in 2015, the overall level remained high and stable.

Hohhot ranked first in 2005; however, its score dropped to fourth in 2015 and fifth in 2018. From 2005 to 2010, Hohhot firmly grasped the opportunities of western development and the national macro-control. Its GDP showed a significant growth and formed a characteristic advantage dominated by the dairy industry, which promoted the rapid development of its economy. Since 2015, the total score of Hohhot dropped from a high level to a medium level, mainly due to restrictions to the location and industrial base. The lagging development of emerging industries, the small scale, and the slow development of foreign trade all led to its weakened economic growth.

Although Nanning is an important economic center along the Beibu Gulf coast and also is a regional international city for the Association of Southeast Asian Nations (ASEAN) countries, its concept of development lags but is close to Guangzhou, which is a developed city. This has led to a large amount of labor outflow. Transportation and other infrastructure requires upgrades, the level of industrial modernization is low, and there is a lack of policy and financial support, which all contribute to the slow economic development. This has led to the overall low ranking of the city.

Guiyang and Xining have maintained a high rate of economic growth, rising from ninth and tenth in 2005 to second and third, respectively, in 2015. However, in 2018, Guiyang dropped to the fifth place and Xining to the tenth. Since 2013, Guiyang has cooperated with Zhongguancun, Beijing, to transform traditional industries on a large scale and build a modern industrial system based on big data. This has effectively promoted its economic growth and shifted the level of new urbanization from medium to high. Due to the foundation of emerging industry is not solid enough, and the support role of real economy is not strong enough, Guiyang’s ranking had a bit of a drop in 2018. From the perspective of dynamic change, the most significant increase in a score at the three considered times was for Xining (an increase of 10.1%). Since the 12th Five-Year Plan, the government of Xining has issued policies, such as financial support for economic restructuring, which has given priority to the development of producer services. Thus, the government has seized the opportunities of the Silk Road Economic Belt to further optimize the investment structure and vigorously develop tertiary industries and cultural services. Therefore, it has achieved rapid economic development. However, compared with its own orientation and other cities along the Belt and Road, Xining’s opening-up level still lags far behind. In 2016–2018, the import and export of Xining continued to plummet, so the ranking of economic scores also declined seriously.

**Fig 5:**
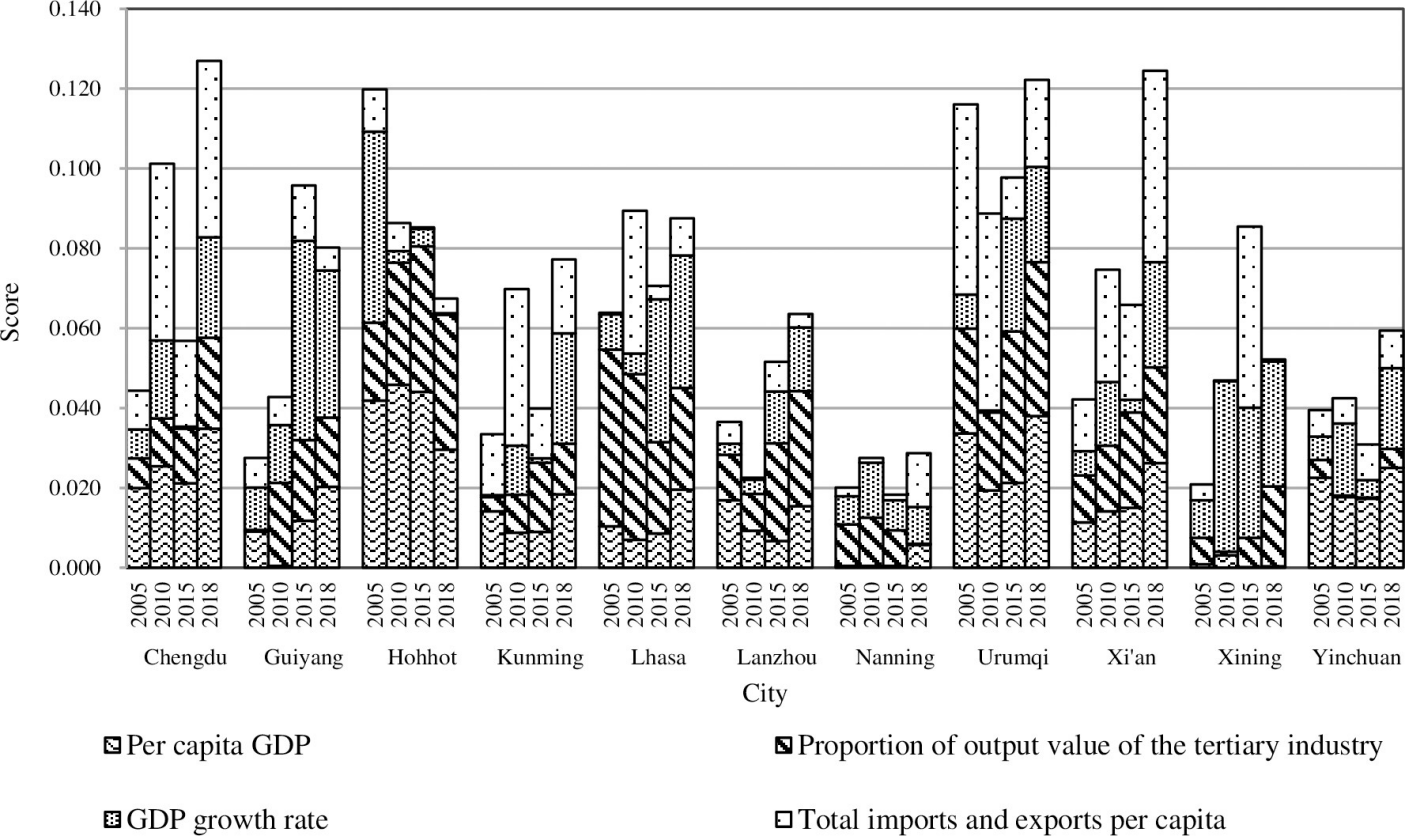
The “economy” of new urbanization in provincial capital cities of Western China.

From 2005 to 2018, the economic development level of each city was extremely uneven with large fluctuations. From the specific indicators (Fig 5), the scores of the GDP growth rate and the total imports and exports per capita varied greatly, which was the main cause of differences in the economic developments. This shows that the current industrial and trade structure has a visible driving effect on economic growth. All these cities should accelerate the transformation of the economic development mode by improving and optimizing the industrial structure to a greater extent and further enhancing economic growth.

#### 4.3.3. Quality of life

During the research period, with the exception of the steady improvement of Nanning, the overall development of the other cities was relatively stable without major fluctuations. However, there are significant differences among the cities. At four research times, Chengdu scored the highest (0.240, 0.215, 0.216, and 0.210, respectively), Xi’an followed closely (0.165, 0.189, 0.176, and 0.128, respectively) and Lhasa and Xining were always at the bottom.

In recent years, Nanning’s tourism and real estate markets have been relatively active, driving the development of the related industries, such as transportation, food, retail, building materials, furniture, and decoration, which has brought significant employment opportunities to the local area and also improved the disposable income of urban residents. The increased wage levels and the accelerated integration of new business models as represented by online sales with the traditional sales model have further stimulated consumption. This has led to a steady improvement in the quality of life for Nanning residents.

Compared with other cities, Chengdu had an absolute leading edge, which was strictly related to its economic development and convenient transportation as an important commercial logistics center and comprehensive transportation hub in China. Xi’an has enacted new policies on the endowment and medical insurance in recent years, which has improved the level of social insurance treatment and covered a wide range of residents’ living securities.

Lhasa and Xining, located in the Qinghai-Tibet plateau, both have a unique physical and geographical environment, which has restricted its economic development and improvements in living standards. With the promotion of urbanization, residents’ living quality of them still has much room for improvement.

**Fig 6:**
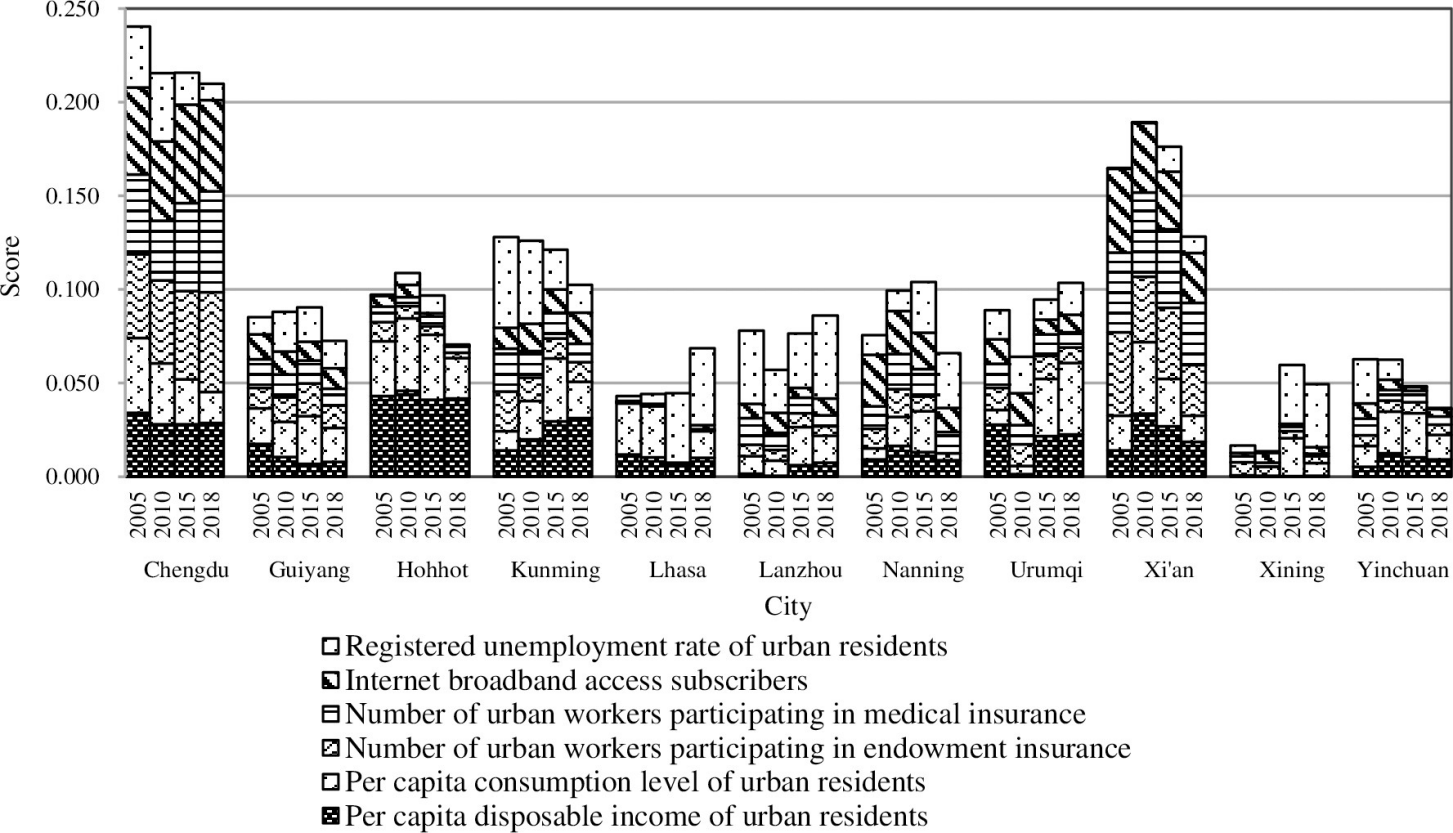
The “quality of life” for new urbanization in the provincial capital cities of Western China.

Based on the specific indicators (Fig 6), the registered unemployment rate of urban residents, the per capita disposable income of urban residents, and the broadband internet subscribers fluctuated significantly, which became the main reason for changes in residents’ quality of life over the different years. In general, the per capita disposable income increases when unemployment falls. Over the past 14 years, the economic development of various cities has absorbed a large amount of the labor market and promoted substantial growth in the per capita economic income. The construction of information technology has transformed the production and lifestyle of residents and promoted the development of new urbanization.

#### 4.3.4. Infrastructure construction

Except for Lanzhou and Lhasa, the scores of other cities showed relatively little change. Lanzhou ranked fourth in 2005 and 2010 (0.076 and 0.078, respectively), but dropped sharply to the last place in 2015 (0.021) and placed the seventh in 2018 (0.076). The other two last place cities in 2005 and 2010 were Nanning and Guiyang (0.018 and 0.017, respectively), and their rankings did not fluctuate much over the three time nodes. In 2005 and 2015, Lhasa topped the list (0.135 and 0.096, respectively) but dropped to fifth place in 2010 (0.071) and 2018 (0.088). Urumqi ranked third in 2005 (0.095), rose to first place in 2010 (0.098), but then fell to sixth place in 2015 (0.065) and rose to fourth in 2018 (0.092). During the entire research period, only Yinchuan has been stable in the top three (0.108, 0.089, 0.093, and 0.099, respectively).

The reason for Lanzhou’s declined ranking is that while promoting the urbanization level, it has not coordinated the development of urbanization quality. The city infrastructure needed repairing, and the “semi-urbanization problem” of the agricultural transfer population was prominent. Besides, the demand for urban public transport exceeded the supply, causing the scores of Nanning and Guiyang to be low.

Yinchuan, which was steadily ranked, has set the goal of the “two best” cities since 2005. That is the best cities for entrepreneurship and livability in Western China. The levels for infrastructure construction and the urban living environment have been greatly improved. In particular, the government has attached great importance to the construction of digital libraries. The owned public library collection per 100 people of Yinchuan has been ranked first in the western provincial capitals for some time. It is noted that of these cities, only Kunming’s ranking constantly improved. This is mainly since the Kunming municipal government has always focused on the construction of infrastructure and has continuously increased its investments.

**Fig 7:**
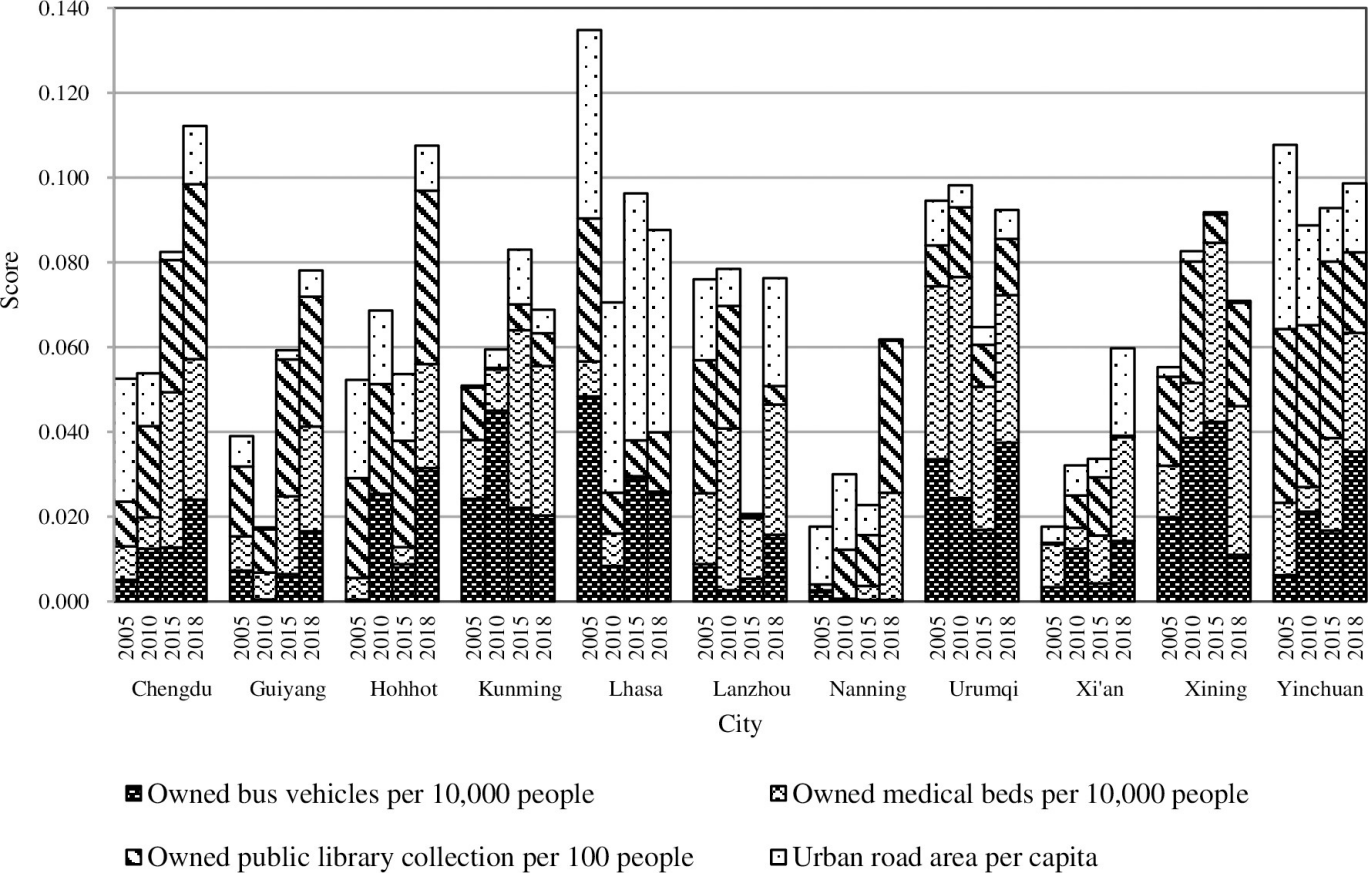
The “infrastructure” of new urbanization in the provincial capital cities of Western China.

According to the specific indicators (Fig 7), the scores of the owned bus vehicles per 10,000 people and the urban road area per capita of different cities were significantly different. This has become the most important factor affecting the differences in infrastructure construction projects between cities. In 2005, due to the lack of permanent urban population data for Lhasa, the indicator scores were calculated according to the household registration population, which were relatively small, and resulted in inflated calculation results. In addition, Lhasa has been continuously increasing investments in infrastructure and key urban projects in recent years, resulting in significant changes in the outlook of the city. However, the backward infrastructure construction is still the main bottleneck that restricts Lhasa and even Tibet’s economic and social development. In 2010, Urumqi invested 500 million Yuan in urban infrastructure maintenance and construction, and the city’s appearance was completely changed. Also, Urumqi’s score soared in 2010, but there was a lack of continuous investment in the follow-up and the ranking was overtaken by other cities. In future urban construction processes, all cities should pay attention to the quality of urban development, promote the orderly equalization of the basic public services, and improve the comprehensive carrying capacity of cities.

#### 4.3.5. Resources and environment

The overall score for the cities in terms of resources and environment shows large fluctuations. Each city at the four time points showed varying degrees of fluctuation, and the top three cities were never the same. In 2005, 2015, and 2018, the highest scores were in Kunming (0.143, 0.156, and 0.154, respectively) but were ranked fourth in 2010. In 2010, the highest score was in Chengdu (0.173), which ranked third in 2005, sixth in 2015, and fourth in 2018, respectively. Hohhot scored fourth in 2005 but fell to sixth and eighth place in 2010 and 2015, respectively, while rose to second in 2018. Yinchuan rose from seventh place in 2005 to third place in 2010 but fell to fifth place in 2015 and 2018. After a relatively smooth development, Xi’an finally rose to third place in 2015, but dropped to eighth in 2018. Although these increases have been attributed to the coordination of the urbanization process with the resources and environment, the above results reflect that economically underdeveloped areas cannot fully consider the development of new urbanization.

It can be seen that Kunming, which enjoys the reputation of being the “Spring City”, has unique advantages in resources and environment. In 2003, Kunming completed the construction of an automatic air quality monitoring system. By the end of 2015, the proportion of good air quality days in the city had remained above 90%. However, in 2010, due to increases in the urban construction land per capita, a negative index, and a reduction in the proportion of harmless treated garbage were caused by the contradiction between the increasing population and garbage per capita per day and the single garbage disposal mode in Kunming. Therefore, its score for resources and the environment declined slightly. Since 2010, Kunming has vigorously carried out urbanization throughout the region, and the area used for urban construction has increased significantly, resulting in a significant increase in the per capita area. Subsequently, the government strengthened the approval process of land for construction projects to control the excessive growth of construction land. During the 12th Five-Year Plan period, the government of Kunming put forward the “8185 Industrial Cultivation and Promotion Plan” to build a plateau lakeside eco-city. Great progress has been made in the economy and ecological construction of Kunming, and the total score of new urbanization was promoted from medium to high in 2015.

In 2010, the green coverage region in the built-up area of Chengdu reached 17,964.71 hectares, and the green coverage rate in the built-up area of Chengdu (39.43%) was higher than the national average (38.22%), further realizing the construction goal of the national ecological garden city. The environmentally sound management rate of domestic waste and the domestic sewage treatment rate in 2010 increased significantly compared to 2005. All these contributed to Chengdu’s first-place ranking in 2010. However, in 2015, due to significant air pollution in Chengdu, the good urban air quality rate was lower at only 58.63%, which decreased Chengdu’s comprehensive score. After 2017, Chengdu issued a number of air governance policies to strengthen the efforts to control haze and improve air quality, so its ranking of resources and environment increased in 2018.

From 2005 to 2015, the main reason Hohhot’s score declined year over year is that its urban air quality has been declining. Hohhot has developed rapidly in recent years, and the urban population and the number of cars have greatly increased, causing the urban air pollution problem to become more serious. Since 2016, taking the 70th anniversary of the founding of the autonomous region as an opportunity, Hohhot has carried out a sustained and in-depth urban environmental remediation action. In 2018, the environment of Hohhot has been significantly improved, and the ranking has also increased remarkably.

The proportion of good air quality days is the main factor that influences the fluctuations in the ranking of Yinchuan. Since 2005, the environmental quality of Yinchuan has steadily improved to meet the goal of being a national environmental protection exemplary city. However, since 2013, 74 key cities, such as Yinchuan, have begun to implement new national air quality standards, which require adding PM2.5, ozone, and carbon monoxide as three new indicators. This altered metric has caused Yinchuan’s air quality to decrease accordingly.

The continuous rise of Xi’an’s score is related to its green coverage rate in built-up areas, the increased proportion of harmless treated garbage, and the increased wastewater treatment rate. Since the 11th Five-Year Plan, Xi’an has eliminated a large number of significantly polluted construction projects, increased investments in environmental protection, increased urban green areas, and effectively enhanced the overall image of the city by implementing three major measures. These are structural adjustments, environmental infrastructure construction, industrial pollution control projects, and the legal means to manage the environment. However, in recent years, severe air pollution has once again restricted score of resources and environment in Xi'an, and the government still needs to strengthen pollution control in the future.

**Fig 8:**
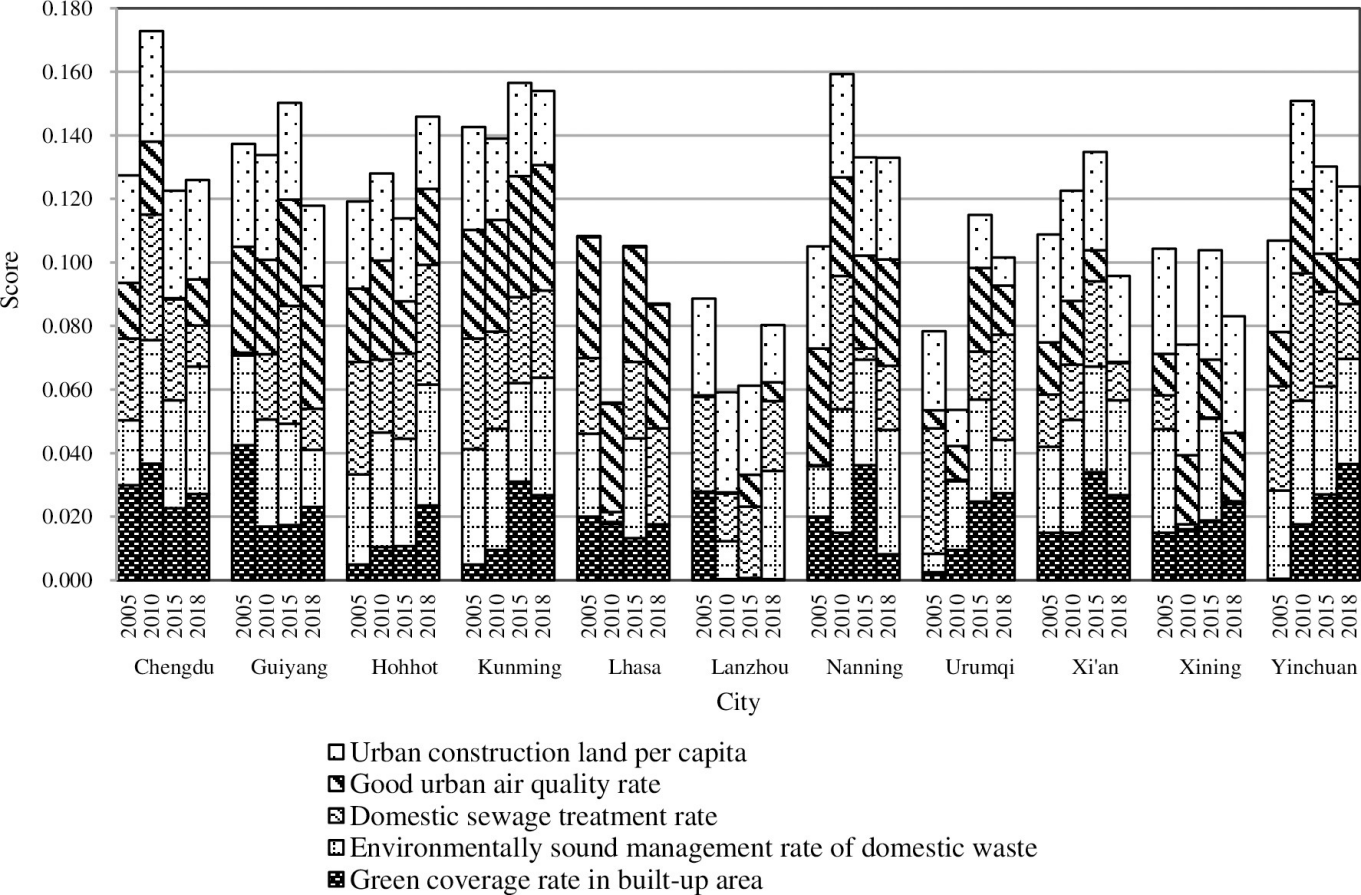
The “resources and environment” of new urbanization in provincial capital cities of Western China.

The specific indicators (Fig 8) show that the scores of the green coverage rate in built-up areas, the proportion of harmless treated garbage, and the good air quality days in cities differ greatly. This indicates that these have become the main factors that affect the differences in the resources and environment of cities over different years. In the future, the process of implementing strategies of new urbanization implies that cities take the construction of green towns as an entry point to address the contradictions between urbanization and the ecological environment while continuing to promote the development of new urbanization.

#### 4.3.6. Urban and rural harmonious development

With the exception of individual cities having fluctuations due to inconsistent data sources, the overall ranking of most cities was relatively stable. Urumqi ranked first in 2005 and 2010 (0.075 and 0.078, respectively) and declined to second place in 2015 (0.073) and 2018 (0.080). Yinchuan ranked second in 2005 (0.055), and continuously declined to fifth place in 2010 (0.032) and 2018 (0.038), and seventh place in 2015 (0.030). Chengdu ranked third in 2005 and 2010 (0.048 and 0.036, respectively), and then rose to first in 2015 and 2018 (0.075 and 0.089, respectively). It is noted that Nanning’s rank lowered in 2005, 2010 and 2015 (0.022, 0.009, and 0.001, respectively), but it ranked fourth in 2018 (0.046).

In recent years, Urumqi has intensified the overall development of urban and rural areas and increased its support for agricultural policies. The income gap between urban and rural areas has been reduced by abolishing agricultural taxes, increasing agricultural subsidies, vigorously promoting new rural cooperative medical care, and introducing new rural old-age insurance. With the implementation of the western development strategy, the economy of Yinchuan has maintained its rapid development. However, because agricultural labor productivity is far lower than that of a typical industry, the income of urban residents is far higher than rural residents, and the income gap between urban and rural areas has increased significantly. As a result, its ranking continues to decline. However, Chengdu continues to rise and topped the list with 0.075 in 2015. One of the reasons is that it took the lead in implementing reforms of the rural property rights system and created a farmland protection fund for the entire country. These policies promoted the orderly circulation of rural capital and increased property income for farmers.

Nanning has a unique location that promotes the rapid development of its economy. In recent years, the incomes of urban and rural residents have increased significantly. However, due to the lack of pillar industries and the weak function of urban services, its role as the central city is insufficient, especially for the surrounding rural areas. Therefore, the gap between urban and rural developments has been large. In 2015, the operating income of rural residents was reduced due to climate change, the rising costs of agricultural materials, and increased land rent, which further widened the income gap between urban and rural residents. After 2016, Nanning has greatly developed characteristic agriculture and e-commerce, which realized the increase of farmers' income and the upgrading of agriculture.

The other three cities of Xining, Lhasa, and Hohhot had rankings that fluctuated greatly. Xining ranked tenth in 2005 but rose to second in 2010 (0.022 and 0.052, respectively). Since 2009, the government of Xining has implemented a series of policies to benefit agriculture, strengthen the construction of agricultural infrastructure, and develop efficient and characteristic agriculture. All of these effectively promoted the development of agriculture and the growth of farmers’ incomes. As a result, the income gap between urban and rural residents was largely reduced. However, with adjustments in agricultural products lagging behind the changes in demand and other factors, some agricultural products appear to produce more than the demand in stages. Thus, the increased rate of farmers’ income reduces, which makes the gap between urban and rural development widen and dropped Xining to ninth in 2015 (0.014), placed seventh in 2018 (0.023), little different from 2015. Lhasa ranked last in 2005 and 2010 and rose to third in 2015 (0.001, 0.004, and 0.055, respectively). This was also thanks to the local government’s continued increase in policy implementation to benefit agriculture. While the harmonious development of urban and rural areas in Hohhot is consistent with the trend of the economy, from 2005 to 2015, Hohhot ranked from fourth to eighth, and ranked sixth in 2018. When the economy improves, the net income of farmers increases and vice versa. While the income of urban residents remains at a relatively stable level over the same period, the income gap widens and causes the ranking to decline.

**Fig 9:**
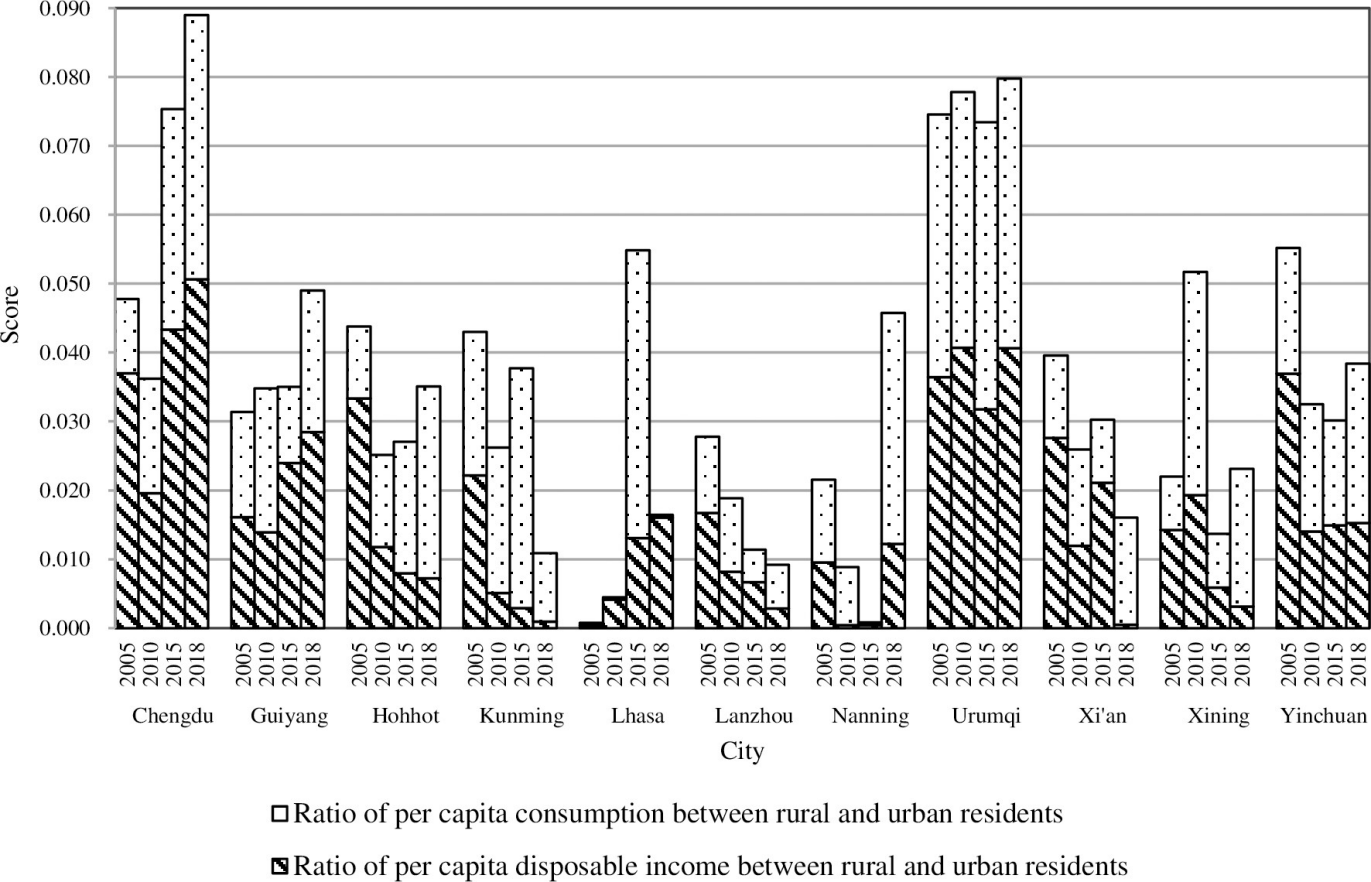
The “urban-rural planning” of new urbanization in provincial capital cities of Western China.

As shown in Fig 9, there are two indicators with relatively significant differences among the cities. These are the ratio of the per capita disposable income between rural and urban residents and the ratio of per capita consumption between rural and urban residents. There are also large differences between these two indicators within specific cities. In 2005 and 2010, over half of the western capitals had a lower consumption ratio than income ratio. However, the urban consumption ratio for all the cities in 2015 and 2018 were higher than the income ratio, significantly reducing the growth. This means that although the income of rural residents increased, the cost of living had risen faster, and the income growth rate was lower than that for urban residents. Therefore, the gap between urban and rural areas has an expanding trend.

To narrow the gap between the rich and poor in urban and rural areas and promote the development of the rural economy, municipal governments should take active measures to realize the integration of urban and rural development. This includes increasing support for rural economies, expanding employment for farmers, guiding rational consumption, accelerating the transformation of urban-rural dual structure, promoting reforms for the household registration system and the land system and the social security system, and others.

## 5. Conclusions and discussion

This research utilized the new urbanization level integrated measuring metric system according to our connotation requirement for new urbanization pathways. This work comprehensively evaluated the urbanization development level in all directions with multiple indexes, which further revealed the significant differences in populations, economic development, residential living quality, infrastructure, and resources and environment for the overall urban and rural developments in provincial capital cities of Western China. And the policy implications were also analyzed correspondingly. The major results are summarized as follows.

1. From 2005 to 2018, the quality of new urbanization was constantly improving, with the quality of life for residents and resources and the environment being the two main driving forces. However, the levels for 11 provincial capitals in Western China showed distinctively uneven developments in the different regions. Chengdu, Kunming, and Xi’an were much better than the other considered cities. In addition, the western capital cities all had a low internal coordination degree for urban development.
2. The factors that influenced the single new urbanization level of each city vary across the 14 years of the study. It was found that the six driving factors have remarkably different evolving characteristics. Compared with the other four single factors, the economic development and resources and environment have large fluctuations. This shows that the economic model of the western provincial capitals needs to be transformed, and the construction of green urbanization is still facing great pressure. In addition, the quality of life is often linked to the local economy, the level of infrastructure is directly related to investments from the local government, and the gap between urban and rural areas is widening. While changes in population development are relatively stable, the ranking of individual factors for each city is different. In the future, new urbanization construction in each city should be emphasized to focus on improving the relatively weak content. At the same time, we should stress the “people-oriented” principle, consider the urbanization quality, reduce the development gap between rural and urban areas, and establish a new concept for the coordinated development between cities and the economy, society, and environment.
3. The settlement policy and reform of the household registration system have alleviated the brain drain to some extent. Economic development depends on Western Development, the Belt and Road, and local policies of economic structure adjustment. The residents' income has been improved and the government continues to issue social security policies, both of which have improved the quality of life for local residents. The improvement of urban infrastructure and living environment cannot be separated from the governments' attention to infrastructure construction and urban environmental governance. Government support for agriculture is an important means for farmers to increase their income and narrow the gap with urban areas.
4. This research constitutes an integrated metric system using the entropy method. Thus, the results are objective and scientific while comprehensively and accurately reflecting the new urbanization development level of western provincial capitals. Meanwhile, the approach combines the GIS Space Analysis Method to reveal their temporal-spatial characteristic. And this work also takes policy implications into account. However, the dynamic analysis of the urbanization development level is limited, and we do not compare the situation in the western region to the entire country or other regions to embody the particularity of the west.

## Supporting information

S1 Table Weight of each index. (DOCX)

S2 Table Weight of factor layers. (DOCX)

S3 Table The city score of “population development”. (DOCX)

S4 Table The city score of “economic development”. (DOCX)

S5 Table The city score of “quality of life”. (DOCX)

S6 Table The city score of “infrastructure”. (DOCX)

S7 Table The city score of “resources and environment”. (DOCX)

S8 Table The city score of “urban and rural harmonious development”. (DOCX)
