# Supplementary material for: Evolution characteristics and policy implications of new urbanization in provincial capital cities in Western China
Source: PLoS One. 2020 May 26;15(5):e0233555. doi: 10.1371/journal.pone.0233555 (PMC7250444; doi:10.1371/journal.pone.0233555)
Supplement: S1 Table — (DOCX) [file pone.0233555.s001.docx]

Table 1 Weight of each index

| Year | Permanent resident urbanization rate (X_11_) | Proportion of non-agricultural employees (X_12_) | Number of college students per 10,000 people (X_13_) | Per capita GDP (X_21_) | Proportion of output value of tertiary industries (X_22_) | GDP growth rate (X_23_) | Total imports and exports per capita (X_24_) | Per capita disposable income of urban residents (X_31_) | Per capita consumption level of urban residents (X_32_) | Registered unemployment rate of urban residents (X_33_) | Number of urban workers participating in endowment insurance (X_34_) | Number of urban workers participating in medical insurance (X_35_) | Broadband internet access subscribers (X_36_) |
| --- | --- | --- | --- | --- | --- | --- | --- | --- | --- | --- | --- | --- | --- |
| 2005 | 0.038 | 0.034 | 0.041 | 0.042 | 0.044 | 0.048 | 0.048 | 0.043 | 0.040 | 0.048 | 0.045 | 0.043 | 0.047 |
| 2006 | 0.038 | 0.034 | 0.038 | 0.043 | 0.049 | 0.041 | 0.040 | 0.044 | 0.040 | 0.044 | 0.044 | 0.042 | 0.047 |
| 2007 | 0.039 | 0.035 | 0.039 | 0.046 | 0.046 | 0.040 | 0.044 | 0.043 | 0.045 | 0.041 | 0.044 | 0.043 | 0.048 |
| 2008 | 0.039 | 0.034 | 0.040 | 0.050 | 0.040 | 0.037 | 0.048 | 0.044 | 0.041 | 0.044 | 0.043 | 0.043 | 0.060 |
| 2009 | 0.039 | 0.034 | 0.041 | 0.048 | 0.042 | 0.036 | 0.045 | 0.046 | 0.041 | 0.042 | 0.045 | 0.045 | 0.058 |
| 2010 | 0.039 | 0.034 | 0.043 | 0.046 | 0.042 | 0.043 | 0.049 | 0.046 | 0.039 | 0.044 | 0.044 | 0.045 | 0.042 |
| 2011 | 0.039 | 0.035 | 0.039 | 0.047 | 0.040 | 0.038 | 0.050 | 0.048 | 0.040 | 0.041 | 0.047 | 0.047 | 0.046 |
| 2012 | 0.038 | 0.034 | 0.040 | 0.045 | 0.038 | 0.044 | 0.052 | 0.048 | 0.049 | 0.040 | 0.048 | 0.047 | 0.045 |
| 2013 | 0.038 | 0.034 | 0.045 | 0.044 | 0.040 | 0.049 | 0.050 | 0.045 | 0.041 | 0.038 | 0.048 | 0.047 | 0.047 |
| 2014 | 0.040 | 0.035 | 0.042 | 0.042 | 0.039 | 0.047 | 0.047 | 0.043 | 0.039 | 0.047 | 0.049 | 0.048 | 0.048 |
| 2015 | 0.038 | 0.034 | 0.042 | 0.044 | 0.038 | 0.050 | 0.045 | 0.041 | 0.035 | 0.037 | 0.047 | 0.047 | 0.053 |
| 2016 | 0.039 | 0.036 | 0.041 | 0.044 | 0.041 | 0.038 | 0.048 | 0.043 | 0.037 | 0.043 | 0.048 | 0.050 | 0.058 |
| 2018 | 0.039 | 0.035 | 0.043 | 0.038 | 0.038 | 0.037 | 0.048 | 0.042 | 0.038 | 0.053 | 0.054 | 0.049 | 0.044 |

Continued Table 1

| Year | Owned bus vehicles per 10,000 people (X_41_) | Owned medical beds per 10,000 people (X_42_) | Owned public library collection per 100 people (X_43_) | Urban road area per capita (X_44_) | Urban construction land per capita (X_51_) | Green coverage rate in built-up area (X_52_) | Proportion of harmless treated garbage (X_53_) | Wastewater treatment rate (X_54_) | Proportion of good air quality days in cities (X_55_) | Ratio of per capita disposable income between rural and urban residents (X_61_) | Ratio of per capita consumption between rural and urban residents (X_62_) |
| --- | --- | --- | --- | --- | --- | --- | --- | --- | --- | --- | --- |
| 2005 | 0.048 | 0.041 | 0.041 | 0.044 | 0.034 | 0.043 | 0.036 | 0.039 | 0.038 | 0.037 | 0.038 |
| 2006 | 0.048 | 0.042 | 0.041 | 0.053 | 0.034 | 0.040 | 0.040 | 0.043 | 0.036 | 0.040 | 0.042 |
| 2007 | 0.050 | 0.042 | 0.043 | 0.047 | 0.035 | 0.041 | 0.036 | 0.040 | 0.038 | 0.040 | 0.038 |
| 2008 | 0.043 | 0.045 | 0.040 | 0.043 | 0.035 | 0.037 | 0.037 | 0.040 | 0.039 | 0.043 | 0.038 |
| 2009 | 0.044 | 0.040 | 0.040 | 0.046 | 0.035 | 0.035 | 0.038 | 0.040 | 0.037 | 0.044 | 0.040 |
| 2010 | 0.045 | 0.052 | 0.038 | 0.045 | 0.035 | 0.037 | 0.039 | 0.042 | 0.035 | 0.041 | 0.037 |
| 2011 | 0.041 | 0.048 | 0.042 | 0.042 | 0.036 | 0.039 | 0.038 | 0.039 | 0.036 | 0.041 | 0.043 |
| 2012 | 0.037 | 0.048 | 0.039 | 0.039 | 0.035 | 0.039 | 0.041 | 0.038 | 0.038 | 0.043 | 0.036 |
| 2013 | 0.048 | 0.046 | 0.039 | 0.041 | 0.035 | 0.036 | 0.035 | 0.037 | 0.037 | 0.043 | 0.040 |
| 2014 | 0.041 | 0.043 | 0.044 | 0.042 | 0.036 | 0.036 | 0.035 | 0.037 | 0.039 | 0.042 | 0.039 |
| 2015 | 0.042 | 0.042 | 0.042 | 0.058 | 0.035 | 0.036 | 0.034 | 0.037 | 0.038 | 0.043 | 0.042 |
| 2016 | 0.040 | 0.037 | 0.040 | 0.042 | 0.037 | 0.037 | 0.035 | 0.037 | 0.041 | 0.049 | 0.040 |
| 2018 | 0.038 | 0.035 | 0.041 | 0.048 | 0.037 | 0.037 | 0.04 | 0.038 | 0.039 | 0.051 | 0.039 |
