# Supplementary material for: Evolution characteristics and policy implications of new urbanization in provincial capital cities in Western China
Source: PLoS One. 2020 May 26;15(5):e0233555. doi: 10.1371/journal.pone.0233555 (PMC7250444; doi:10.1371/journal.pone.0233555)
Supplement: S2 Table — (DOCX) [file pone.0233555.s002.docx]

Table 2 Weight of factor layers

| Year | Population development (X1) | Economic development(X2) | Quality of life (X3) | Infrastructure (X_4_) | Resources and environment (X5) | Urban and rural harmonious development (X6) |
| --- | --- | --- | --- | --- | --- | --- |
| 2005 | 0.113 | 0.182 | 0.265 | 0.175 | 0.190 | 0.075 |
| 2006 | 0.109 | 0.173 | 0.261 | 0.183 | 0.192 | 0.082 |
| 2007 | 0.113 | 0.176 | 0.263 | 0.182 | 0.190 | 0.078 |
| 2008 | 0.113 | 0.175 | 0.273 | 0.170 | 0.188 | 0.081 |
| 2009 | 0.114 | 0.171 | 0.276 | 0.170 | 0.186 | 0.083 |
| 2010 | 0.115 | 0.179 | 0.260 | 0.180 | 0.187 | 0.078 |
| 2011 | 0.112 | 0.175 | 0.269 | 0.172 | 0.188 | 0.084 |
| 2012 | 0.113 | 0.179 | 0.276 | 0.162 | 0.191 | 0.079 |
| 2013 | 0.117 | 0.182 | 0.266 | 0.174 | 0.178 | 0.083 |
| 2014 | 0.118 | 0.175 | 0.273 | 0.170 | 0.183 | 0.081 |
| 2015 | 0.114 | 0.177 | 0.260 | 0.184 | 0.180 | 0.085 |
| 2016 | 0.116 | 0.170 | 0.279 | 0.159 | 0.188 | 0.088 |
| 2018 | 0.117 | 0.161 | 0.280 | 0.162 | 0.191 | 0.090 |
