# Supplementary material for: Evolution characteristics and policy implications of new urbanization in provincial capital cities in Western China
Source: PLoS One. 2020 May 26;15(5):e0233555. doi: 10.1371/journal.pone.0233555 (PMC7250444; doi:10.1371/journal.pone.0233555)
Supplement: S3 Table — (DOCX) [file pone.0233555.s003.docx]

Table 3 The city score of “population development”

| City | 2005 | 2006 | 2007 | 2008 | 2009 | 2010 | 2011 | 2012 | 2013 | 2014 | 2015 | 2016 | 2018 |
| --- | --- | --- | --- | --- | --- | --- | --- | --- | --- | --- | --- | --- | --- |
| Chengdu | 0.083 | 0.087 | 0.086 | 0.084 | 0.084 | 0.072 | 0.075 | 0.078 | 0.070 | 0.074 | 0.073 | 0.071 | 0.071 |
| Kunming | 0.076 | 0.077 | 0.075 | 0.075 | 0.076 | 0.067 | 0.078 | 0.082 | 0.073 | 0.078 | 0.079 | 0.082 | 0.085 |
| Guiyang | 0.103 | 0.105 | 0.097 | 0.093 | 0.095 | 0.086 | 0.086 | 0.085 | 0.084 | 0.092 | 0.089 | 0.094 | 0.094 |
| Xi'an | 0.112 | 0.108 | 0.112 | 0.109 | 0.109 | 0.107 | 0.103 | 0.102 | 0.091 | 0.096 | 0.094 | 0.097 | 0.087 |
| Lanzhou | 0.099 | 0.090 | 0.089 | 0.093 | 0.102 | 0.108 | 0.112 | 0.112 | 0.117 | 0.117 | 0.114 | 0.116 | 0.115 |
| Xining | 0.064 | 0.063 | 0.064 | 0.061 | 0.061 | 0.058 | 0.059 | 0.061 | 0.060 | 0.061 | 0.059 | 0.062 | 0.064 |
| Lhasa | 0.007 | 0.011 | 0.007 | 0.005 | 0.004 | 0.004 | 0.016 | 0.007 | 0.004 | 0.006 | 0.004 | 0.013 | 0.003 |
| Urumchi | 0.052 | 0.053 | 0.048 | 0.053 | 0.051 | 0.042 | 0.051 | 0.046 | 0.039 | 0.042 | 0.046 | 0.045 | 0.046 |
| Yinchuan | 0.070 | 0.079 | 0.079 | 0.083 | 0.082 | 0.062 | 0.073 | 0.075 | 0.070 | 0.074 | 0.073 | 0.073 | 0.075 |
| Hohhot | 0.090 | 0.095 | 0.093 | 0.097 | 0.098 | 0.089 | 0.092 | 0.085 | 0.079 | 0.082 | 0.074 | 0.082 | 0.078 |
| Nanning | 0.048 | 0.054 | 0.058 | 0.064 | 0.059 | 0.052 | 0.057 | 0.054 | 0.056 | 0.058 | 0.053 | 0.062 | 0.063 |
