# Supplementary material for: Evolution characteristics and policy implications of new urbanization in provincial capital cities in Western China
Source: PLoS One. 2020 May 26;15(5):e0233555. doi: 10.1371/journal.pone.0233555 (PMC7250444; doi:10.1371/journal.pone.0233555)
Supplement: S4 Table — (DOCX) [file pone.0233555.s004.docx]

Table 4 The city score of “economic development”

| City | 2005 | 2006 | 2007 | 2008 | 2009 | 2010 | 2011 | 2012 | 2013 | 2014 | 2015 | 2016 | 2018 |
| --- | --- | --- | --- | --- | --- | --- | --- | --- | --- | --- | --- | --- | --- |
| Chengdu | 0.044 | 0.064 | 0.069 | 0.070 | 0.102 | 0.101 | 0.107 | 0.078 | 0.070 | 0.091 | 0.057 | 0.088 | 0.127 |
| Kunming | 0.033 | 0.047 | 0.050 | 0.067 | 0.062 | 0.070 | 0.069 | 0.063 | 0.073 | 0.059 | 0.040 | 0.062 | 0.077 |
| Guiyang | 0.027 | 0.038 | 0.050 | 0.045 | 0.061 | 0.043 | 0.071 | 0.068 | 0.092 | 0.095 | 0.096 | 0.077 | 0.080 |
| Xi'an | 0.042 | 0.048 | 0.059 | 0.083 | 0.089 | 0.075 | 0.074 | 0.052 | 0.057 | 0.087 | 0.066 | 0.113 | 0.125 |
| Lanzhou | 0.037 | 0.033 | 0.030 | 0.037 | 0.032 | 0.023 | 0.047 | 0.030 | 0.056 | 0.059 | 0.052 | 0.068 | 0.064 |
| Xining | 0.021 | 0.030 | 0.028 | 0.037 | 0.034 | 0.047 | 0.031 | 0.037 | 0.039 | 0.054 | 0.085 | 0.044 | 0.052 |
| Lhasa | 0.064 | 0.089 | 0.077 | 0.043 | 0.062 | 0.089 | 0.109 | 0.109 | 0.112 | 0.101 | 0.071 | 0.076 | 0.088 |
| Urumchi | 0.116 | 0.113 | 0.118 | 0.136 | 0.090 | 0.089 | 0.123 | 0.124 | 0.129 | 0.098 | 0.098 | 0.088 | 0.122 |
| Yinchuan | 0.039 | 0.057 | 0.056 | 0.066 | 0.049 | 0.042 | 0.029 | 0.033 | 0.028 | 0.052 | 0.031 | 0.052 | 0.059 |
| Hohhot | 0.120 | 0.112 | 0.119 | 0.100 | 0.121 | 0.086 | 0.088 | 0.080 | 0.085 | 0.082 | 0.085 | 0.099 | 0.067 |
| Nanning | 0.020 | 0.044 | 0.050 | 0.049 | 0.059 | 0.028 | 0.029 | 0.025 | 0.015 | 0.015 | 0.018 | 0.018 | 0.029 |
