# Supplementary material for: Evolution characteristics and policy implications of new urbanization in provincial capital cities in Western China
Source: PLoS One. 2020 May 26;15(5):e0233555. doi: 10.1371/journal.pone.0233555 (PMC7250444; doi:10.1371/journal.pone.0233555)
Supplement: S5 Table — (DOCX) [file pone.0233555.s005.docx]

Table 5 The city score of “quality of life”

| City | 2005 | 2006 | 2007 | 2008 | 2009 | 2010 | 2011 | 2012 | 2013 | 2014 | 2015 | 2016 | 2018 |
| --- | --- | --- | --- | --- | --- | --- | --- | --- | --- | --- | --- | --- | --- |
| Chengdu | 0.240 | 0.242 | 0.247 | 0.193 | 0.178 | 0.215 | 0.221 | 0.216 | 0.216 | 0.223 | 0.216 | 0.218 | 0.210 |
| Kunming | 0.128 | 0.124 | 0.134 | 0.167 | 0.163 | 0.126 | 0.132 | 0.130 | 0.107 | 0.126 | 0.121 | 0.115 | 0.103 |
| Guiyang | 0.085 | 0.084 | 0.092 | 0.076 | 0.079 | 0.088 | 0.088 | 0.085 | 0.090 | 0.085 | 0.091 | 0.087 | 0.073 |
| Xi'an | 0.165 | 0.138 | 0.127 | 0.126 | 0.163 | 0.189 | 0.192 | 0.220 | 0.221 | 0.201 | 0.176 | 0.171 | 0.128 |
| Lanzhou | 0.078 | 0.079 | 0.060 | 0.061 | 0.050 | 0.057 | 0.057 | 0.066 | 0.072 | 0.086 | 0.076 | 0.093 | 0.086 |
| Xining | 0.017 | 0.019 | 0.021 | 0.017 | 0.014 | 0.014 | 0.011 | 0.031 | 0.028 | 0.027 | 0.060 | 0.059 | 0.049 |
| Lhasa | 0.043 | 0.044 | 0.045 | 0.038 | 0.061 | 0.044 | 0.036 | 0.041 | 0.039 | 0.040 | 0.045 | 0.060 | 0.069 |
| Urumchi | 0.089 | 0.080 | 0.073 | 0.051 | 0.045 | 0.064 | 0.057 | 0.047 | 0.061 | 0.081 | 0.094 | 0.109 | 0.103 |
| Yinchuan | 0.063 | 0.071 | 0.064 | 0.070 | 0.069 | 0.062 | 0.060 | 0.045 | 0.041 | 0.053 | 0.048 | 0.047 | 0.037 |
| Hohhot | 0.097 | 0.107 | 0.119 | 0.112 | 0.115 | 0.109 | 0.112 | 0.117 | 0.106 | 0.095 | 0.097 | 0.092 | 0.071 |
| Nanning | 0.076 | 0.074 | 0.064 | 0.081 | 0.086 | 0.099 | 0.105 | 0.099 | 0.103 | 0.109 | 0.104 | 0.077 | 0.066 |
