# Supplementary material for: Evolution characteristics and policy implications of new urbanization in provincial capital cities in Western China
Source: PLoS One. 2020 May 26;15(5):e0233555. doi: 10.1371/journal.pone.0233555 (PMC7250444; doi:10.1371/journal.pone.0233555)
Supplement: S7 Table — (DOCX) [file pone.0233555.s007.docx]

Table 7 The city score of “resources and environment”

| City | 2005 | 2006 | 2007 | 2008 | 2009 | 2010 | 2011 | 2012 | 2013 | 2014 | 2015 | 2016 | 2018 |
| --- | --- | --- | --- | --- | --- | --- | --- | --- | --- | --- | --- | --- | --- |
| Chengdu | 0.127 | 0.137 | 0.160 | 0.158 | 0.133 | 0.173 | 0.163 | 0.131 | 0.138 | 0.132 | 0.123 | 0.138 | 0.126 |
| Kunming | 0.143 | 0.155 | 0.132 | 0.162 | 0.135 | 0.139 | 0.152 | 0.175 | 0.153 | 0.162 | 0.156 | 0.159 | 0.154 |
| Guiyang | 0.137 | 0.128 | 0.142 | 0.136 | 0.123 | 0.134 | 0.167 | 0.156 | 0.145 | 0.146 | 0.150 | 0.154 | 0.118 |
| Xi'an | 0.109 | 0.145 | 0.137 | 0.119 | 0.118 | 0.123 | 0.133 | 0.134 | 0.126 | 0.122 | 0.135 | 0.132 | 0.096 |
| Lanzhou | 0.089 | 0.092 | 0.080 | 0.059 | 0.073 | 0.059 | 0.080 | 0.081 | 0.088 | 0.065 | 0.061 | 0.077 | 0.080 |
| Xining | 0.104 | 0.120 | 0.111 | 0.124 | 0.083 | 0.074 | 0.102 | 0.098 | 0.105 | 0.110 | 0.104 | 0.149 | 0.083 |
| Lhasa | 0.108 | 0.101 | 0.109 | 0.103 | 0.086 | 0.056 | 0.085 | 0.088 | 0.094 | 0.113 | 0.105 | 0.091 | 0.087 |
| Urumchi | 0.078 | 0.075 | 0.064 | 0.050 | 0.065 | 0.054 | 0.069 | 0.052 | 0.110 | 0.092 | 0.115 | 0.096 | 0.102 |
| Yinchuan | 0.107 | 0.117 | 0.119 | 0.154 | 0.167 | 0.151 | 0.160 | 0.159 | 0.150 | 0.139 | 0.130 | 0.140 | 0.124 |
| Hohhot | 0.119 | 0.117 | 0.113 | 0.123 | 0.123 | 0.128 | 0.122 | 0.116 | 0.102 | 0.110 | 0.114 | 0.132 | 0.146 |
| Nanning | 0.105 | 0.106 | 0.123 | 0.117 | 0.137 | 0.159 | 0.134 | 0.098 | 0.142 | 0.152 | 0.133 | 0.138 | 0.133 |
