# Supplementary material for: Evolution characteristics and policy implications of new urbanization in provincial capital cities in Western China
Source: PLoS One. 2020 May 26;15(5):e0233555. doi: 10.1371/journal.pone.0233555 (PMC7250444; doi:10.1371/journal.pone.0233555)
Supplement: S8 Table — (DOCX) [file pone.0233555.s008.docx]

Table 8 The city score of “urban and rural harmonious development”

| City | 2005 | 2006 | 2007 | 2008 | 2009 | 2010 | 2011 | 2012 | 2013 | 2014 | 2015 | 2016 | 2018 |
| --- | --- | --- | --- | --- | --- | --- | --- | --- | --- | --- | --- | --- | --- |
| Chengdu | 0.048 | 0.036 | 0.042 | 0.038 | 0.032 | 0.036 | 0.033 | 0.037 | 0.041 | 0.049 | 0.075 | 0.081 | 0.089 |
| Kunming | 0.043 | 0.037 | 0.046 | 0.036 | 0.024 | 0.026 | 0.030 | 0.022 | 0.034 | 0.031 | 0.038 | 0.017 | 0.011 |
| Guiyang | 0.031 | 0.014 | 0.012 | 0.029 | 0.026 | 0.035 | 0.026 | 0.032 | 0.028 | 0.047 | 0.035 | 0.044 | 0.049 |
| Xi'an | 0.040 | 0.026 | 0.035 | 0.028 | 0.022 | 0.026 | 0.029 | 0.040 | 0.025 | 0.033 | 0.030 | 0.037 | 0.016 |
| Lanzhou | 0.028 | 0.015 | 0.023 | 0.018 | 0.019 | 0.019 | 0.013 | 0.019 | 0.021 | 0.021 | 0.011 | 0.010 | 0.009 |
| Xining | 0.022 | 0.019 | 0.030 | 0.040 | 0.039 | 0.052 | 0.060 | 0.057 | 0.062 | 0.076 | 0.014 | 0.013 | 0.023 |
| Lhasa | 0.001 | 0.001 | 0.008 | 0.001 | 0.001 | 0.004 | 0.009 | 0.010 | 0.012 | 0.025 | 0.055 | 0.014 | 0.016 |
| Urumchi | 0.075 | 0.082 | 0.078 | 0.081 | 0.083 | 0.078 | 0.084 | 0.079 | 0.083 | 0.078 | 0.073 | 0.077 | 0.080 |
| Yinchuan | 0.055 | 0.037 | 0.040 | 0.030 | 0.028 | 0.032 | 0.031 | 0.032 | 0.040 | 0.040 | 0.030 | 0.036 | 0.038 |
| Hohhot | 0.044 | 0.033 | 0.034 | 0.024 | 0.024 | 0.025 | 0.018 | 0.025 | 0.024 | 0.040 | 0.027 | 0.030 | 0.035 |
| Nanning | 0.022 | 0.015 | 0.019 | 0.012 | 0.007 | 0.009 | 0.007 | 0.014 | 0.004 | 0.001 | 0.001 | 0.043 | 0.046 |
